# Connecting the Dots in the Neuroglobin-Protein Interaction Network of an Unstressed and Ferroptotic Cell Death Neuroblastoma Model

**DOI:** 10.3390/cells8080873

**Published:** 2019-08-11

**Authors:** Zoë P. Van Acker, Geert A. Van Raemdonck, Emilie Logie, Sara I. Van Acker, Geert Baggerman, Wim Vanden Berghe, Peter Ponsaerts, Sylvia Dewilde

**Affiliations:** 1 Laboratory of Protein Science, Proteomics and Epigenetic Signaling (PPES), Department of Biomedical Sciences, University of Antwerp, 2610 Wilrijk, Belgium; 2 Centre for Proteomics (CFP), University of Antwerp, 2610 Wilrijk, Belgium; 3 Department of Ophthalmology, Visual Optics and Visual Rehabilitation, Faculty of Medicine and Health Sciences, University of Antwerp, 2610 Wilrijk, Belgium; 4 Flemish Institute for Technological Research (VITO), 2400 Mol, Belgium; 5 Laboratory of Experimental Hematology (LEH), Faculty of Medicine and Health Sciences, University of Antwerp, 2610 Wilrijk, Belgium

**Keywords:** neuroglobin, ferroptosis, protein-interaction network, neuroprotection

## Abstract

Neuroglobin is a heme protein of which increased levels provide neuroprotection against amyloid proteinopathy and hemorrhagic damage. These cellular stressors involve the promotion of ferroptosis—an iron-dependent, lipid peroxide-accreting form of cell death. Hence, we questioned whether neuroglobin could oppose ferroptosis initiation. We detected human neuroglobin (hNgb)-EGFP-expressing SH-SY5Y cells to be significantly more resistant to ferroptosis induction, identifying 0.68-fold less cell death. To elucidate the underlying pathways, this study investigated hNgb-protein interactions with a Co-IP-MS/MS approach both under a physiological and a ferroptotic condition. hNgb binds to proteins of the cellular iron metabolism (e.g., RPL15 and PCBP3) in an unstressed condition and shows an elevated binding ratio towards cell death-linked proteins, such as HNRNPA3, FAM120A, and ABRAXAS2, under ferroptotic stress. Our data also reveal a constitutive interaction between hNgb and the longevity-associated heterodimer XRCC5/XRCC6. Disentangling the involvement of hNgb and its binding partners in cellular processes, using Ingenuity Pathway Analysis, resulted in the integration of hNgb in the ubiquitination pathway, mTOR signaling, 14-3-3-mediated signaling, and the glycolysis cascade. We also detected a previously unknown strong link with motor neuropathies. Hence, this study contributes to the elucidation of neuroglobin’s involvement in cellular mechanisms that provide neuroprotection and the upkeep of homeostasis.

## 1. Introduction

Apoptotic cell death after an injury is a well-established phenomenon. It contributes to the pathological progression of neurodegenerative diseases, e.g., Alzheimer’s disease (AD). However, the pathology cannot be reduced to an aggregation-induced apoptosis cascade. AD-affected brains show signs, which do not correspond with apoptosis characteristics, e.g., glial inflammation [1] and iron metabolism dysregulation [2]. The latter may cause reactive oxygen and nitrogen species (ROS/RNS) levels to rise, leading to the observed increase in lipid peroxidation of membranes in AD brains [3]. Interestingly, these features correspond to a newly discovered form of oxidative, caspase-independent, iron-linked cell death—ferroptosis [4]. Key to ferroptotic cell death is the accretion of lipid peroxide. In line with the latter, it has been found that the abrogation of antioxidant defenses is linked to ferroptosis initiation, i.e., the attenuation of the activity of the lipid repair enzyme glutathione peroxidase 4 (GPX4) and the cystine-glutamate antiporter system x_c_^−^ [4]. Ferroptosis further induces ER stress that mediates p53-independent PUMA expression, causing some synergistic interaction with the apoptosis process [5]. However, in contrast to apoptosis, the process of ferroptotic cell death also includes the release of damage-associated molecular patterns that cause immune-cell infiltration in ferroptotic areas [6]. Given the above, it may be of no surprise that ferroptosis not only plays a detrimental role in the context of AD [7] but also in other human pathologies [8], such as intracerebral hemorrhages [9], ischemic stroke [10], acute kidney failure [11,12], periventricular leukomalacia [12], and other neurodegenerative pathologies, such as Parkinson’s disease (PD) [13] and Huntington’s disease [12].

Pharmacologically, ferroptosis can be induced by three classes of molecules [14]. The first class inhibits GPX4 by reducing glutathione levels (e.g., erastin, SRS13-60, and buthionine sulfoximine). The second class directly inhibits GPX4 itself (e.g., RSL3, DPI7, and DPI10), and molecules of the third group work through the generation of ROS (e.g., Sorafenib and artemisinin derivatives) [14]. In this study, we used erastin. This compound founded our knowledge of the ferroptosis process. It was identified in a high-throughput screening that aimed to detect new anti-cancer agents [15]. Interestingly, this small molecule caused a new form of cell death with distinctive cellular characteristics, such as a decreased mitochondrial volume and the reduction or vanishing of mitochondrial crista [15]. It was found that erastin works through a gain-of-function mechanism involving voltage-dependent anion channel2/3, altering ion selectivity, and initiating mitochondria-driven oxidative stress [16]. Detrimental accretion of ROS is further promoted by erastin’s blocking of the cystine-glutamate antiporter system x_c_^−^, which is also involved in the formation of antioxidant glutathione [4]. Erastin signaling is detected to be reliant on the RAS–RAF–MEK cascade as well [16]. These erastin-induced ferroptosis processes have been described in different human-derived neuroblastoma cell lines, including SH-SY5Y cells; the model system of this paper [17].

Neuroglobin (Ngb) has been identified as a cytoprotective protein within the nervous system. This heme protein belongs to the globin family, scavenges ROS/RNS, and augments the threshold for apoptosis initiation (reviewed in [18]). We also recently correlated the expression levels of Ngb with those of repressor element 1-silencing transcription factor (REST/NRSF) [19,20]. Of interest, REST/NRSF represses genes that endorse cell death, oxidative stress, and amyloid β-protein toxicity [21]. Given upregulated Ngb levels to be found neuroprotective in pathologies that also involve ferroptotic cell death, e.g., hemorrhages and AD [22], we questioned whether Ngb could indeed effectuate protection against erastin-induced ferroptosis. To elucidate the underlying pathways, we further investigated Ngb-protein interactions both under a physiological and an erastin-stressed condition. To date, a small number of studies has reported experimental evidence for protein interactions with Ngb [23,24,25,26]. Most prominent are Ngb’s interactions with cytochrome molecules, B5 and C [24,25], and 14-3-3 adaptor proteins [23], providing a direct link to the apoptosis process. However, a yeast two-hybrid screen was most often used, entailing only limited coverage between studies and a lack of (sub)cellular context [25,26]. Moreover, no Co-IP mass spectrometry approach has been used yet for the discovery of Ngb-protein interactions in a human-derived neuronal system.

We detected the ‘cell death and survival, cellular development, cellular growth, and proliferation’ pathway to be the pathway with the highest correlation with Ngb-protein interactions. As its name already reveals, the pathway substantiated our hypothesis that human neuroglobin (hNgb) functions reach further than the obstruction of apoptosis initiation. hNgb binds 78 kDa glucose-regulated protein (HSPA5), ubiquitin carboxyl-terminal hydrolase isozyme L1 (UCHL1), and 14-3-3 protein zeta/delta (YWHAZ). All three bind in turn to the amyloid precursor protein (APP). Hence, these pathway axes might be essential in the observed lowering of the cellular amyloid load in AD mouse models that transgenically overexpress Ngb [22]. The network also revealed a connection between Ngb and ‘nuclear factor (erythroid-derived 2)-like 2’ (NRF2) through their common interactor RNA-binding protein EWS (EWSR1). NRF2 transcription was significantly increased in our Ngb-EGFP SH-SY5Y cells, but not in EGFP control cells under ferroptotic stress. Such a link between Ngb and the main regulator of enzymes of the antioxidant glutathione pathway, and the cellular oxidative stress response in general, is of interest [27,28,29]. It broadens Ngb’s anti-oxidative function beyond its intrinsic scavenger function [30].

## 2. Materials and Methods

### 2.1. Stable Cell Line Generation

Neuroblastoma SH-SY5Y cells (ATCC CRL-2266) were cultured in the complete growth medium, containing a 1:1 mixture of Eagle’s Minimum Essential Medium and F12 medium (Gibco, Gent, Belgium). The mix was further supplemented with 10% fetal calf serum, 1 mM sodium pyruvate, 0.1 mM non-essential amino acids, and 1.5 g/L sodium bicarbonate (Gibco).

The hNgb-EGFP fusion protein was generated with the coding region of human Ngb (NM_021257.3), which was ligated in the pEGFP-N1 vector (Clontech, Saint-Germain-en-Laye, France), as described previously [31]. The sequence of the resultant vector was verified using Sanger sequencing (Supplementary Figure S1). The correct vector was transfected in SH-SY5Y cells, using nucleofector technology, according to the manufacturer’s instructions (Lonza, Verviers, Belgium). Briefly, 1.5 × 10^6^ SH-SY5Y cells were resuspended in 100 μl nucleofector solution and combined with 2 μg of the vector. Subsequently, the cell/DNA suspension was inserted in the Nucleofector™ 2b Device (Lonza), and the G-004 program was executed. From twenty-four hours post-transfection, cells were cultured in complete growth medium containing 600 µg/ml Geneticin (G418) Sulfate (Fisher Scientific, Gent, Belgium). Selection medium was replaced every 3 to 4 days for the next 4 weeks. The vector-control cell line was generated accordingly with an empty pEGFP-N1 vector.

### 2.2. Flow-Cytometric Phenotyping and Analysis of Cell Viability

For intracellular staining of Ngb, hNgb-EGFP and EGFP control SH-SY5Y cells were fixed (2% paraformaldehyde), made permeable (ethanol −20 °C), and stained subsequently with anti-Ngb (Protein tech., Manchester United Kingdom, 13499-1-AP, 1:100) and anti-rabbit IgG, F(ab’)2 frag.Cy3 (Sigma-Aldrich, Overijse, Belgium, C2306, 1:200) for 1 h each.

Staurosporine (apoptosis inducer, Enzo Life Sciences, Antwerp, Belgium) and erastin (ferroptosis inducer, Selleckchem, Munich, Germany) were dissolved in dimethylsulfoxide to a 1 mM stock solution and stored at −80 °C. Before use, staurosporine and erastin were further diluted in complete growth medium to experimental concentrations. Cell death was analyzed by staining with propidium iodide (PI) (ROTH, Keerbergen, Belgium) and annexin V-PE (Biovision, Kampenhout, Belgium); the latter being included for detection of staurosporine-induced apoptosis. Annexin V^+^/PI^−^ cells were considered to be early apoptotic, while late apoptotic and necrotic cells were characterized as annexin V^+^/PI^+^ and annexin V^−^/PI^+^, respectively.

**Figure 1:**
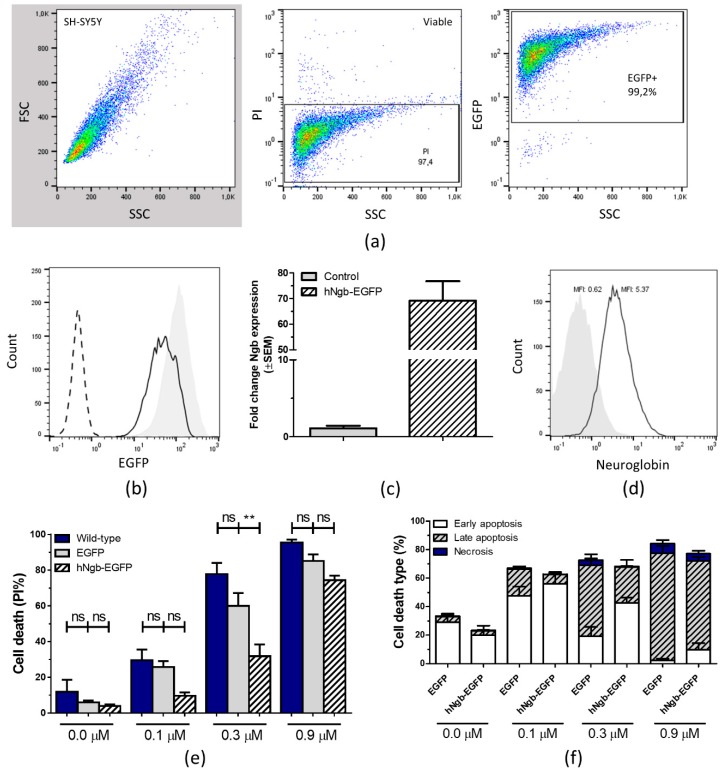
Human neuroglobin (hNgb)-EGFP SH-SY5Y cell line validation and anti-apoptotic characteristics. (**a**) Gating strategy for flow cytometric analysis. Initial gating was performed on propidium iodide (PI) and side scatter (SSC) plot, eliminating dead cells. Within the viable cell gate, EGFP+ (or Cy3+) signals were identified. (**b**) Representative histogram profiles of EGFP-positivity in the wild-type (0% EGFP+, dashed line), EGFP control (99.2% EGFP+, gray shaded), and hNgb-EGFP (99.6% EGFP+, black line) SH-SY5Y cell lines, as determined from the gated viable fraction (PI-negative cells). (**c**) Bar graphs illustrate hNgb transcript levels in unstressed control and hNgb-EGFP SH-SY5Y cells (n = 4), as normalized to the global mean. (**d**) Histogram overlays show a representative example of the mean fluorescent intensity (MFI) after intracellular staining of hNgb in SH-SY5Y cells, transfected with EGFP (gray shaded, MFI 0.62) or hNgb-EGFP (black line, MFI 5.37). (**e/f**) Flow-cytometric data of SH-SY5Y cells subjected to 24 h of staurosporine-induced cell death (n = 5–6). Data are depicted as the mean ± SEM. (**e**) Total percentage of dead cells, as indicated by PI-positivity. Statistical significance was tested using one-way ANOVA with Bonferroni’s multiple comparisons test; ** *p* < 0.01. (**f**) Subclassification of cell death, i.e., early apoptosis (annexin V+/PI−), late apoptosis (annexin V+/PI+), and necrosis (annexin V−/PI+).

Data were acquired on an EPICS XL-MCL Flow Cytometer (Beckman Coulter, Suarlée, Belgium) and analyzed using FlowJo software (v10; Treestar, Ashland, OR, USA). The cytometer-stopping gate was set up for 10,000 events. The applied gating strategy can be consulted in Figure 1a.

### 2.3. Lipid Peroxidation

Levels of malondialdehyde (MDA), an end product of lipid peroxidation, were studied using the lipid peroxidation (MDA) assay kit (Sigma-Aldrich), according to the manufacturer’s instructions. Briefly, 10E6 cells were homogenized in MDA Lysis Buffer. The reaction with thiobarbituric acid was carried out at 95 °C for 60 min, generating a colorimetric (532 nm) MDA-thiobarbituric acid adduct. Concentrations of MDA were calculated from a standard curve and corrected for sample amount.

### 2.4. IncuCyte Kinetic Monitoring of Cell Death

The specificity of erastin-induced cell death was assessed on an IncuCyte system (Essen Bioscience, Ann Arbor, MI, USA). Cells were pre-treated for 2 h with different cell death inhibitors: radical-trapping inhibitor Fer1 (Xcess Biosciences, San Diego, CA, USA, 053224, 10 µM), caspase inhibitor Z-VAD-FMK (Bachem, Bubendorf, Switzerland, N-1510, 50 µM), or RIP1 kinase inhibitor Nec1 (Calbiochem, Overijse, Belgium, 480065, 20 µM) together with 250 nM IncuCyte® Cytotox Red (Essen Bioscience), to detect dead cells. Erastin (50 µM) was added at time-point 0. Fluorescent and phase-contrast images were taken every 2 h in the IncuCyte system for 24 h and analyzed with the Incucyte Analysis Software. The percentage of nuclear Cytotox Red signal (dead cells) was normalized to the percentage of cellular confluency mask value. The data represent the mean fold increase in cell death from a representative time course experiment performed in triplicate.

### 2.5. RNA Extraction and Reverse Transcription Quantitative PCR

Total cellular RNA was extracted from control and hNgb-EGFP SH-SY5Y cells with the PureLink RNA Mini Kit (Life Technologies, Merelbeke, Belgium), according to the manufacturer’s instructions. Obtained RNA concentrations were determined on a NanoDrop (Thermo Scientific, Gent, Belgium), and 1 µg of the isolated RNA was used for first-strand cDNA synthesis. cDNA was generated using the GoScript Reverse Transcription System (Promega, AJ Leiden, The Netherlands), following the manufacturer’s recommendations for random primed synthesis (0.5 µg random primers/reaction). RT-qPCR was conducted on a StepOnePlus system (Life Technologies). Each reaction contained 7.5 ng cDNA along with Power SYBR Green PCR Master Mix (Thermo Scientific) and 150 nM of each primer (Table S1). *ACTB* and *B2M* housekeeping values were used as invariant endogenous controls. Cycling parameters included 95 °C for 2 min, then 40 cycles of 95 °C for 15 s and 60 °C for 1 min. Data analysis was performed with qBase+ (Version 3.0, BioGazelle, Gent, Belgium).

### 2.6. Protein-Protein Interactions

#### 2.6.1. Cell Culturing

Stably transfected hNgb-EGFP and EGFP control SH-SY5Y cells were grown in the complete growth medium. For SILAC (stable isotope labeling with amino acid in cell cultures) experiments, the hNgb-EGFP SH-SY5Y cells were maintained in DMEM:F12 (1:1) medium for SILAC (Thermo Fisher Scientific), supplemented with 2 mM L-glutamine (Gibco), 10% dialyzed, heat-inactivated fetal bovine serum (Sigma-Aldrich), and 100 U/ml penicillin-streptomycin (Gibco). L-arginine (Arg0) and L-lysine (Lys0) (‘Light’), ^13^C_6_^14^N_4_-L-arginine (Arg6) and 4,4,5,5-D4-L-lysine (Lys4) (‘Medium’), or ^13^C_6_^15^N_4_-L-arginine (Arg10) and ^13^C_6_^15^N_2_-L-lysine (Lys8) (‘Heavy’) were used for metabolic labeling (0.1 g/L, Thermo Fisher Scientific). In the “reverse” experiment, the labels were moved to the next condition in line as compared to the first run. For the analysis of hNgb-protein interactions under ferroptosis, 50 μM of erastin (Selleckchem) was added to the medium of the labeled cells for the last 24 h of culture.

#### 2.6.2. Co-Immunoprecipitation

Cells were pelleted, resuspended in lysis buffer (10 mM Tris pH 7.5, 100 mM NaCl, 1% NP40, protease inhibitor complete mini-Roche) and further lysed by sonicating 1 min at 70 Hz. For SILAC experiments, the latter was performed after 6 passages (validated incorporation degree of 99.5%). The resulting cell lysate was incubated with the anti-GFP ab290 Clone (Abcam, Cambridge, United Kingdom) for 1 h at 4 °C on a rotating arm. After centrifuging the mixture for 2 min at 181 g (4 °C), the supernatant of the lysate was mixed with a pellet of 20 µl protein G agarose (Roche, Vilvoorde, Belgium), which was pre-incubated with blocking buffer (lysis buffer without protease inhibitors + 2% milk powder). The mixture was kept for 90 min (4 °C) on a rotating arm. After 4 washes with lysis buffer, proteins were eluted by boiling the samples for 3 min in decoupling buffer (4% SDS, 10% β-mercaptoethanol, 0.125M Tris HCL pH 6.8, 20% glycerol). Eluates were transferred to PBS buffer, using 6 wash steps in an Amicon Ultra-0.5 centrifugal device (Merck Millipore, Overijse, Belgium).

#### 2.6.3. Protein Digestion

An amount of 100 µg protein was taken for non-SILAC analysis, or equal amounts of proteins from different SILAC conditions were pooled (total of 100 µg). Rapigest (Waters Corporation, Asse, Belgium) was added to a final concentration of 0.1% (volume). After incubation at 100 °C (5 min), the sample was let to cool down and treated with 8.7 mM tris(2-carboxyethyl)phosphine (TCEP, Thermo Scientific). Proteins were reduced (1 h at 55 °C) and, subsequently, alkylated with 15.6 mM 2-Iodoacetamide (Thermo Scientific) during a 30 min incubation at room temperature protected from light. Acetone precipitation was carried out with 6 volumes of pre-chilled (−20 °C) acetone (Thermo Scientific) and overnight freezing at −20 °C. The obtained protein pellet (8000 g for 10 min at 4 °C) was re-suspended in 100 mM triethylammonium bicarbonate buffer (pH 8.5, Sigma-Aldrich) and overnight incubated with Trypsin Gold (Promega) to a final protease:protein ratio of 1:40 (w/w) at 37 °C. The digest was abrogated by freezing the sample at −20 °C.

#### 2.6.4. LC-MS/MS Analysis

Digested samples were separated by nano reverse phase C18 (RP-C18) chromatography on an Easy-nLC 1000 system (Thermo Scientific) by using an Acclaim C18 PepMap™ 100 column (75 µm × 2 cm, 3 µm particle size) serially connected to an Acclaim PepMap™ RSLC C18 analytical column (50 µm × 15 cm, 2 µm particle size) (Thermo Scientific). For each sample, a total amount of 1 µg of tryptic peptides was loaded on the column. Before loading, digests were diluted in mobile phase A (2% acetonitrile and 0.1% formic acid) at a concentration of 1 µg/10µl and spiked with 20 fmol Glu-1-fibrinopeptide B (GluFib, Protea biosciences, Morgantown, USA) as an internal standard. A linear gradient from 0 to 45% of mobile phase B (100% acetonitrile with 0.1% formic acid) over 50 min followed by a steep increase to 100% mobile phase B in 10 min was applied at a flow rate of 300 nL/min. Liquid chromatography was followed by MS (LC-MS/MS) and was performed on a Q-Exactive Plus mass spectrometer equipped with a nanospray ion source (Thermo Fisher). The high-resolution mass spectrometer was set up in data-dependent acquisition mode where a full MS1 scan (mass range of 350–1850 *m*/*z*) with a resolution of 70,000 was followed by a high energy collision activated dissociation (HCD) MS2 scan (mass range of 100–2000 m/z) at a resolution of 17,500. Peptide ions were selected for further interrogation by tandem MS as the twenty most intense peaks of a full scan mass spectrum. The normalized collision energy used was set at 28%, and a dynamic exclusion list of 20 s was applied.

#### 2.6.5. Mass Spectrometric Data Analysis

Proteome Discoverer 2.1 SP1 (Thermo Scientific) was used to perform database screening against the human SwissProt database (UP000005640, 20,231 entries), according to the Mascot search algorithm (version 2.5.1). A precursor mass tolerance of 10 ppm and a fragment mass tolerance of 0.02 Da were applied. Trypsin was specified as the digesting enzyme, and 2 missed cleavages were allowed. Carbamidomethylation of cysteine residues and SILAC 3-plex (Arg0, Lys0; Arg6, Lys4, and Arg10, Lys8) were defined as fixed modifications, while methionine oxidation was selected as variable modification. Only medium and high confident peptides with a global FDR < 1% based on a target-decoy approach and first ranked peptides were included in the results. In the SILAC 3-plex quantitation workflow, the precursor ion quantifier node was used to calculate abundance ratios between light, medium, and heavy labeled conditions.

Pathway analysis was performed with the Ingenuity Pathway Analysis (IPA) software and database (Ingenuity® Systems, www.ingenuity.com, Redwood City, CA, USA), according to the instructions provided. Only molecules and/or relationships were considered in which ‘species = human’ and ‘confidence = experimentally observed’. All nervous system tissues and cell lines were considered.

### 2.7. Statistical Analysis

GraphPad Prism software v5.0 (San Diego, CA, USA) was used for statistical calculations and artwork. P-values below 0.05 were considered statistically significant. All data are depicted as the mean ± standard error of the mean (SEM).

## 3. Results

### 3.1. Characterization and Validation of a hNgb-EGFP SH-SY5Y Cell Line

SH-SY5Y cells were stably transfected with a pEGFP-N1 vector in which the coding region of hNgb (NM_021257.3) was ligated. We first validated that over 99% of the cells expressed the construct, confirmed as EGFP-positivity on flow cytometry (Figure 1b). The obtained hNgb-EGFP transgenic cell line has (69.23 ± 7.56)-fold increased transcript levels of hNgb (Figure 1c), which results in hNgb protein levels to be about 8-fold above endogenous levels (Figure 1d). Knowing Ngb to possess a well-documented anti-apoptotic property, the functionality of the fusion protein was evaluated as increased resistance against staurosporine-induced cell death (Figure 1e,f). Given the generated cells lines to be overexpression models, we also validated the amount of cell death in untransfected SH-SY5Y cells. Overexpression under the cytomegalovirus (CMV) promoter does not significantly alter cell viability in our neuroblastoma model, with cell death values of EGFP control and wild-type cells being not significantly different from one another (Figure 1e).

### 3.2. hNgb-EGFP SH-SY5Y Cells Have a Higher Threshold for Ferroptosis Initiation

**Figure 2:**
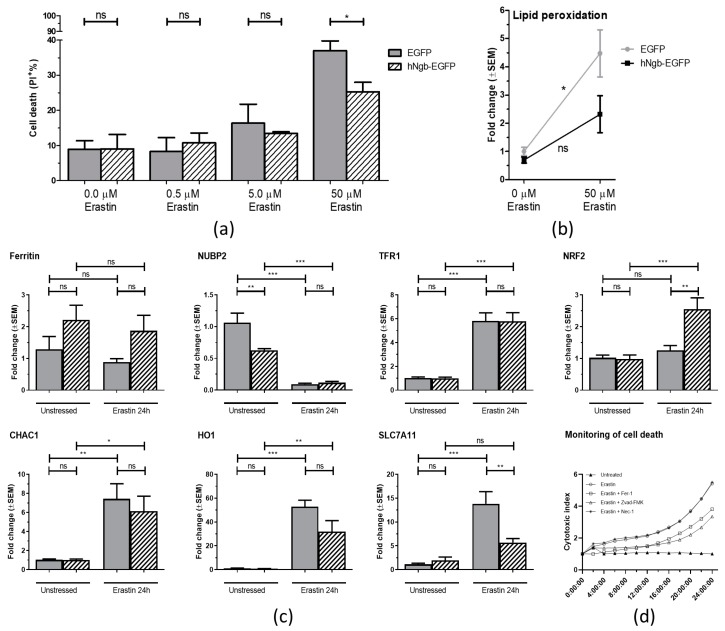
Anti-ferroptotic characteristics of hNgb. (**a/b**) EGFP control and hNgb-EGFP SH-SY5Y cells were treated with erastin for 24 h (n = 3). (**a**) Cell death was determined by flow cytometric analysis of propidium iodide permeability. (**b**) Relative increases of malondialdehyde levels, an end-product of lipid peroxidation. Statistical significance was tested using student’s t-tests; * *p* < 0.05. (**c**) Real-time qPCR data of human glutathione-specific gamma-glutamylcyclotransferase 1 (CHAC1), ferritin, heme oxygenase 1 (HO1), nuclear factor erythroid 2-related factor 2 (NRF2), cytosolic Fe-S cluster assembly factor NUBP2 (NUBP2), cystine/glutamate transporter (SLC7A11), and transferrin receptor protein 1 (TFR1), and expression levels in EGFP control and hNgb-EGFP SH-SY5Y cells. Cells were unstressed or stressed with 50 µM erastin for 24 h (n = 6/group). Data are depicted as the mean ± SEM. Statistical significance was tested using one-way ANOVA with Bonferroni posthoc test; * *p* < 0.05, ** *p* < 0.01, *** *p* < 0.001. (**d**) Validation of erastin (50 µM) cell death specificity, using cell death inhibitors: Fer1 (Ferroptosis inhibitor, 10 µM), Z-VAD-FMK (Apoptosis inhibitor, 50 µM), and Nec1 (Necroptosis inhibitor, 20 µM). Cell death was detected using the IncuCyte® Cytotox Red (250 nM) reagent and normalized to the cell area. The data represent the mean fold increase in cell death from a representative time course experiment performed in triplicate.

Having assessed the validity of the model, we next sought to study the potential neuroprotective role of hNgb towards ferroptosis induction. To this end, the hNgb-EGFP cells were cultured in increasing concentrations of erastin for 24 h. At a 50 µM erastin concentration, the EGFP control cell line displays a significantly higher percentage of cell death (37.03 ± 2.76)% than the hNgb-overexpressing SH-SY5Y cells (25.30 ± 2.69)% (Figure 2a). In addition, malondialdehyde levels are 4.48-fold higher in erastin-stressed EGFP control cells as compared to the unstressed condition. On the contrary, the upregulation seen in hNgb-EGFP cells does not reach statistical significance (Figure 2b).

The specificity of the erastin-induced stress and cell death was validated as the rescue by Fer1 and Z-VAD-FMK (Figure 2d, [5]), as well as by the increase of ferroptosis-associated transcripts: *CHAC1*, *HO1*, *SLC7A11*, and *TFR1* (Figure 2c, [13,32,33]). Interestingly, *SLC7A11* expression is not significantly increased in hNgb-EGFP cells (Figure 2c), possibly contributing to the lower cell death levels that are observed (Figure 2a). Other altered expressions supporting the elevated threshold for ferroptosis-associated neuronal damage in hNgb-EGFP cells include those of *NUBP2.* Its transcript levels are higher in the unstressed EGFP control cells as compared to the unstressed hNgb-EGFP cells (Figure 2c). A 24 h incubation with 50 μM erastin lowers both levels to an equivalent degree of 0.093±0.013 and 0.116±0.019 the baseline, respectively (Figure 2c). Transcription of *ferritin* is overall higher in hNgb-EGFP cells as compared to the controls, though these increases did not reach significance. Both cell lines further increase their *TFR1* expression ~5.8-fold upon addition of erastin to the culture medium (Figure 2c). Lastly, we detected a (2.55 ± 0.36)-fold increase in the expression of neuroprotective *NRF2* in erastin-stressed hNgb-EGFP cells, an upsurge not seen in EGFP control cells (Figure 2c).

### 3.3. Protein Interactors of hNgb in a Non-Stress Condition are Enriched in the ‘Cell Death and Survival, Cellular Development, Cellular Growth, and Proliferation’ Network

**Figure 3:**
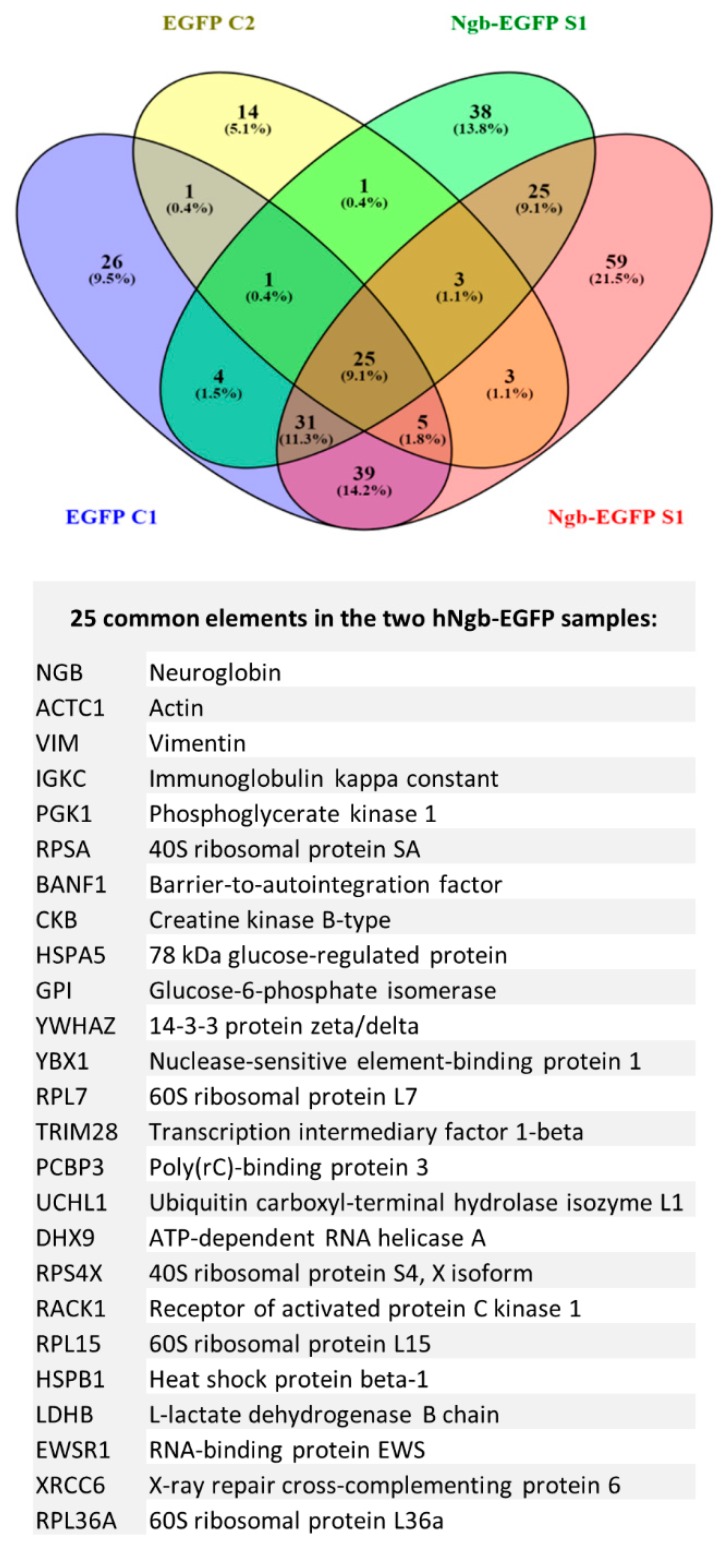
hNgb protein interactors in a non-stress condition. Two independent co-immunoprecipitation (Co-IP) experiments were performed, followed by an LC-MS/MS analysis. We detected 122 proteins in the hNgb-EGFP samples, of which 25 could be validated in both analyses. Proteins identified in the EGFP control samples 1 and/or 2 were considered to be false positives and were not considered for subsequent analyses. The Venn diagram was created by using the online tool Venny 2.1 (http://bioinfogp.cnb.csic.es/tools/venny/).

Two parallel Co-IP experiments were performed in which we looked for specific (hNgb-EGFP cells) and aspecific (EGFP control cells) protein interactions, using an LC/MS-MS approach. We detected 122 proteins in the hNgb-EGFP samples, which were not detected in the EGFP control samples (Figure 3). Aside from hNgb, we identified 24 proteins in both hNgb-EGFP samples, validating the interaction. Supplementary Table S2 comprises a summary of the different described functions of each of the 24 proteins.

**Figure 4:**
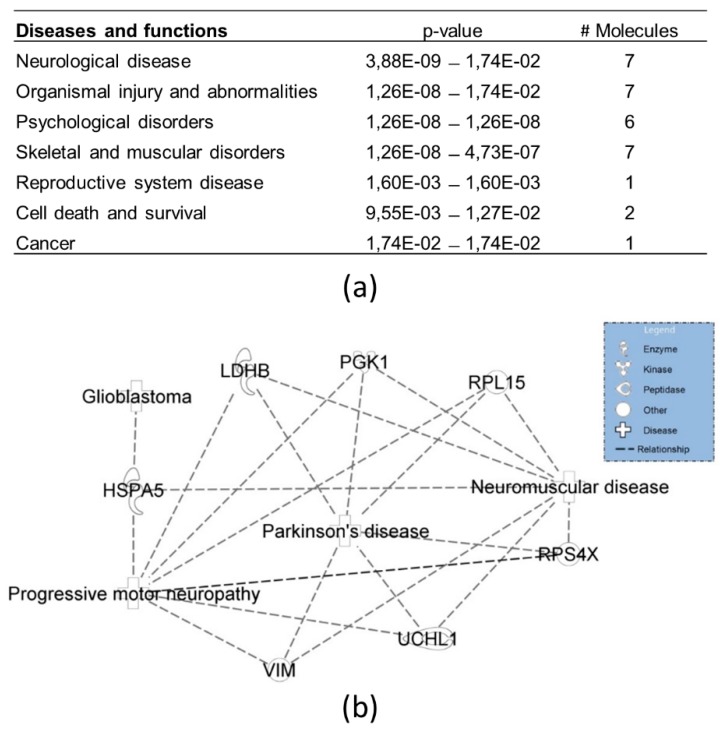
Top diseases and biological functions related to the hNgb protein interactors in a non-stress condition. (**a**) Network analysis by Ingenuity Pathway Analysis reveals the 24 specific hNgb interactors to be specifically linked to neurological diseases and organismal injury and abnormalities. The proteins involved are: 78 kDa glucose-regulated protein (HSPA5), L-lactate dehydrogenase B chain (LDHB), phosphoglycerate kinase 1 (PGK1), 60S ribosomal protein L15 (RPL15), 40S ribosomal protein S4 X isoform (RPS4X), ubiquitin carboxyl-terminal hydrolase isozyme L1 (UCHL1), and vimentin (VIM). (**b**) Function annotations of the neurological diseases network show a clear link to motor neuropathies.

Of note, cellular functions of RPL15 and PCBP3 can be linked to the iron metabolism, thus also establishing a link with ferroptosis on the protein level. While other functions of the 24 proteins can be linked to mechanisms as neuronal sprouting (YWHAZ, GPI, RACK1, VIM), proteostasis (RPS4X, HSPA5, GPI, RACK1, UCHL1, HSPB1), or cell division and differentiation (YWHAZ, RPS4X, TRIM28, YBX1, PGK1, RACK1, XRCC6, EWSR1) by sight; we also performed an Ingenuity Pathway Analysis (IPA) on these 24 binders. Functional analysis recognized the seven diseases and/or biological functions that can be most closely associated with the inserted proteins (Figure 4a). Interestingly, a strong correlation was detected with neurological diseases, which could be brought back to especially motor neuropathies (Figure 4b).

**Figure 5:**
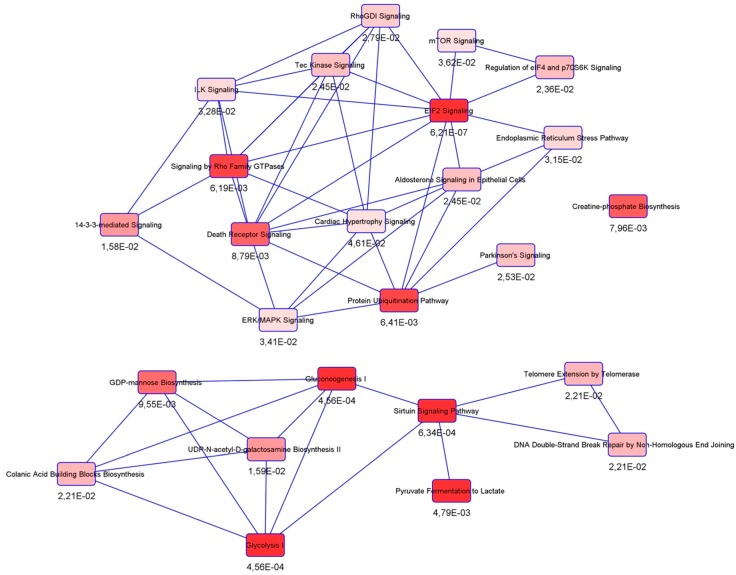
Canonical pathways linked to hNgb binders in a non-stress condition. Ingenuity Pathway Analysis on the 24 specific Ngb-binders reveals connections with inter alia the protein ubiquitination pathway, mTOR signaling, ER stress pathway, 14-3-3-mediated signaling, and glycolysis.

**Figure 6:**
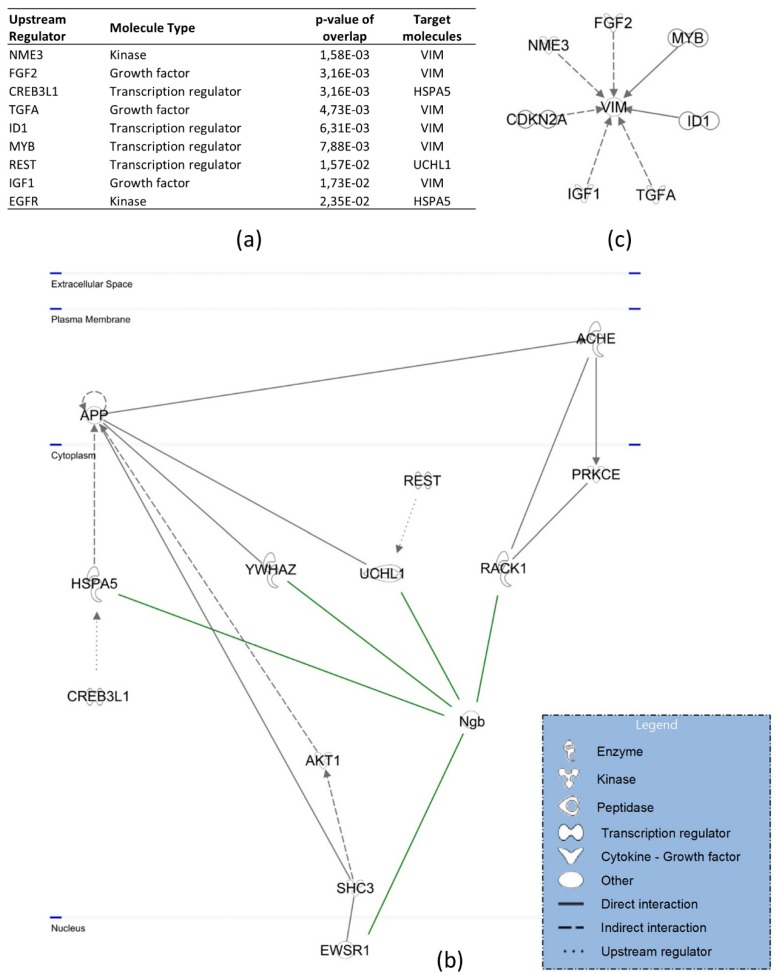
Upstream regulators and signaling cascades linked to the hNgb protein interactors in a non-stress condition. (**a**) The top predicted upstream regulators of hNgb-linked pathways: nucleoside diphosphate kinase 3 (NME3), fibroblast growth factor 2 (FGF2), cyclic AMP-responsive element-binding protein 3-like protein 1 (CREB3L1), transforming growth factor-alpha (TGFA), DNA-binding protein inhibitor ID-1 (ID1), transcriptional activator Myb (MYB), RE1-silencing transcription factor (REST), insulin-like growth factor I (IGF1), and epidermal growth factor receptor (EGFR). (**b**) The ‘cell death and survival, cellular development, cellular growth, and proliferation’ pathway network shows the highest connectivity with hNgb-protein interactions. The latter are indicated as green in the figure. (**c**) Vimentin does not only interact with hNgb but has been identified as the hNgb-interactor with the highest number of predicted connections itself. These provide an indirect link with/influence on hNgb functions.

Canonical pathway analysis detected connections with inter alia the protein ubiquitination pathway, mTOR signaling, the ER stress pathway, 14-3-3-mediated signaling, and the glycolysis cascade (Figure 5). As for upstream regulators, the majority has been found to interact with hNgb-interacting vimentin (Figure 6a,c). The other three (*CREB3L1*, *REST/NRSF*, *EGFR*) are linked to the cell death and survival pathway, in which hNgb interacts with multiple proteins (Figure 6b).

**Figure 7:**
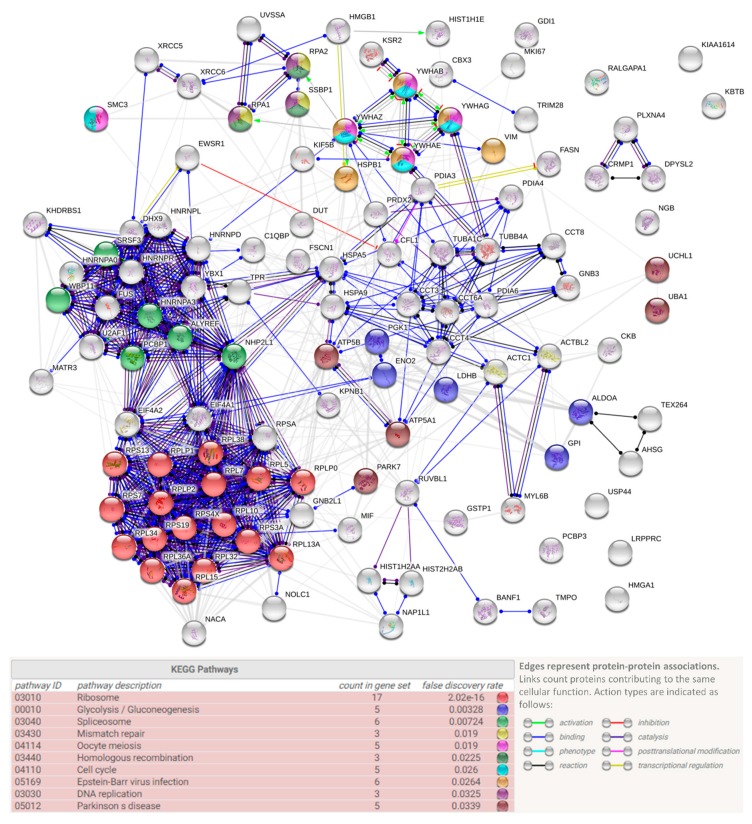
hNgb protein interaction network. Representation of protein-protein associations of the hNgb-specific binders identified in either hNgb-EGFP S1 or S2. The interaction network was obtained using the STRING database (http://string-db.org/) and obtained a PPI enrichment *p*-value of <1.0 × 10^−16^. Proteins belonging to functional Kyoto Encyclopedia of Genes and Genomes (KEGG) pathway enrichments are indicated in the corresponding color. Hits associated with functions of the 24 validated proteins could provide an extra connection between hNgb and processes, such as cell proliferation (MKI67), cytoskeleton organization and axon guidance (CRMP1, CFL1, DPYSL2, PLXNA4), the cell energy metabolism (ALDOA, LRPPRC, ENO2), and proteostasis or protein folding (USP44, CCT4, PDIA6, CCT6A, CCT8, UBA1). Of interest regarding the link between hNgb and ferroptosis, hNgb was also identified to interact with GSTP1 in one sample. Another reactive oxygen species (ROS) metabolism protein (PRDX2) and ATP synthase subunits (ATP5A1, ATP5B) were identified to bind hNgb as well.

Ninety-seven of the hNgb-binders were, however, only detected in one of both hNgb-EGFP samples; 38 and 59 proteins were solely identified in, respectively, hNgb-EGFP sample 1 and 2 (Figure 3). The reason as to why they were not detected in both samples could be due to their rare or weak interaction with hNgb (Table S3). It concerns, for example, neurodegenerative-associated proteins (FUS, PARK7) and proteins belonging to cellular processes which are also associated with functions of the 24 validated proteins (Figure 7). When generating a protein-protein association network of all the hNgb-EGFP non-background binders, irrespective of whether they were detected in sample 1 and/or 2, a protein-protein interaction enrichment *p*-value was obtained of < 1.0E-16 (Figure 7). Given the number of edges (734) to be higher as anticipated from a network obtained from a random protein list of the same size (289), this strengthens our hypothesis of hNgb binders to be biologically connected.

### 3.4. SILAC Labeling Shows Ferroptosis-Specific hNgb Interactions

**Figure 8:**
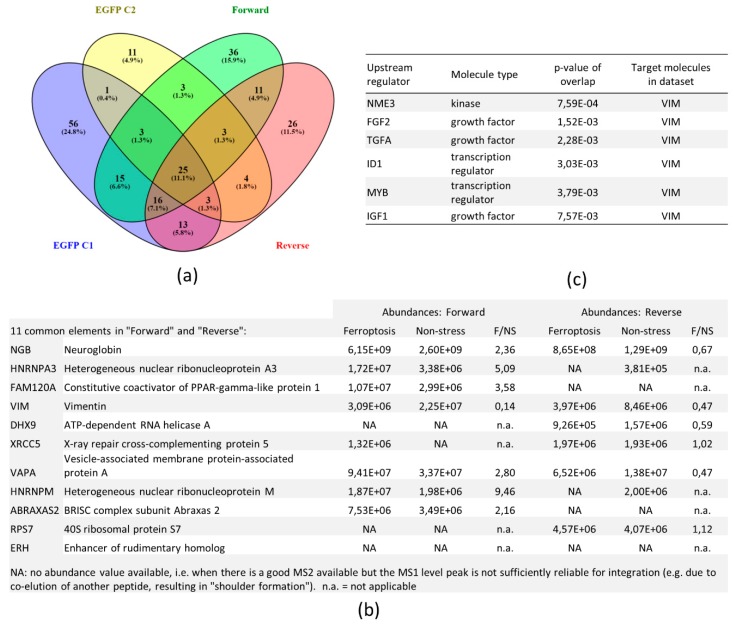
hNgb protein interactors in a ferroptotic condition. (**a**) Venn diagram of the different samples. The forward and reverse samples contain differently labeled proteins of the hNgb-EGFP cells being unstressed or stressed with 50 µM erastin for 24 h. Stable isotope labeling with amino acid in cell cultures (SILAC) labeling was coupled to a Co-IP MS/MS analysis for protein identification. Proteins detected in the EGFP control samples 1 and/or 2 were considered to be background binders and were not included in further analyses. The Venn diagram was created with Venny 2.1 (http://bioinfogp.cnb.csic.es/tools/venny/). (**b**) Abundance values of the ten proteins, which were identified in both the forward and reverse run. Values are represented per condition, and ratios are taken thereof. (**c**) The top predicted upstream regulators of the signaling cascades, which are linked to the 10 identified hNgb-binders.

Stable isotope labeling with amino acid in cell cultures (SILAC) was used to make a distinction between constitutive and ferroptosis-specific hNgb interactions. Since the experiment was repeated after interchanging the SILAC labels between the conditions, the analysis contains two biological replicates (Figure 8a).

To obtain a high specificity of the interactions found, only proteins that were identified in both the forward and the reverse experiment were used. Proteins, which were also identified in the control EGFP samples, were considered to be aspecific binders to either EGFP, the antibody, or agarose beads. Aside from hNgb, ten specific proteins were identified (Figure 8b). This number is lower as one of the unlabeled experiments, which may be attributed to the increased complexity of a SILAC sample. Known biological functions of the ten interactors are listed in Supplementary Table S4. Binding ratios of hNgb and proteins involved in cell death (HNRNPA3, FAM120A, ABRAXAS2) are increased under erastin-stress (Figure 8b). Of note, though not validated, HNRNPA3 was also identified in hNgb-EGFP sample 2 in the unlabeled experiment (Table S3). Popping up in both the unstressed and ferroptotic conditions of the SILAC experiment, this finding underscores our hypothesis that Table S3 could still contain specific binders. In this regard, the 36 proteins only identified in the SILAC forward experiment and the 26 proteins of the reverse experiment (Figure 8a), are listed in Supplementary Table S5.

Concordantly, while XRCC6 was validated as a hNgb interactor in the unlabeled experiment (being present in both hNgb-EGFP samples), the other part of the DNA-repair complex XRCC5/XRCC6 was also identified in the hNgb-EGFP sample 1 of the unlabeled experiment (Table S3). The opposite identification was found in the SILAC experiment, validating the XRCC5 protein with a hit in both the forward and reverse run (Figure 8b) and identifying the XRCC6 protein in the reverse SILAC run but not the forward one (Table S5). However, no significant difference was observed between the interaction abundances of the non-stress and ferroptosis condition (Figure 8b). This categorizes the hNgb-XRCC5/XRCC6 interaction as a constitutive one. hNgb further showes a mean 0.31-fold decrease in interaction abundance with the neurite repair protein vimentin when cells are erastin-stressed. On the contrary, hNgb binding to the axonal targeting protein HNRNPM is increased by 9.46-fold (Figure 8b). Hence, though these interactions with hNgb have been established, it remains to be shown whether hNgb-binding works as an activator or rather inhibitor. As the abundance values of the interaction with VAPA were inconsistent between the forward and reverse experiment, additional validation is warranted to establish the role of this interaction during ferroptosis. Of note, IPA analysis of upstream regulators of hNgb-interactor pathways identified again vimentin as the target molecule in the dataset (Figure 8c).

## 4. Discussion

Since its discovery in 2000 by Burmester and colleagues [34], Ngb, a neuronal-specific heme protein, has been ascribed with neuroprotective functions. Ferrous Ngb inactivates pro-apoptotic cytochrome c at a rate of 2 × 10^7^ M^−1^s^−1^ [24]. Its expression is upregulated during hypoxic events [35] and upsurging Ngb levels entails more favorable outcomes in, for example, the AD pathogenesis and eye pathologies [22,36]. As the alleviations of different pathological characteristics cannot be ascribed to solely the aversion of apoptosis initiation, current data leave a paramount question unanswered; in which cellular pathways is Ngb involved? To investigate Ngb-linked neuroprotective cascades, this study created and validated a transgenic hNgb-EGFP SH-SY5Y cell line for its further use in unraveling Ngb non-stress and stimulus-specific interactions.

We chose erastin as a stressor, knowing ferroptotic cell death to be associated with pathologies in which increased Ngb levels were already described to work neuroprotective [37]. Eight-fold higher endogenous levels of hNgb in our hNgb-EGFP SH-SY5Y cells were indeed ascertained to result in over 10% less cell death as compared to values of EGFP control cells when stressing the cells with 50 µM erastin for 24 h. Hence, this is the first study to describe a widening of the Ngb endogenous neuroprotective actions to ferroptotic cell death. Searching for the underlying cascades that contribute to this promotion of cellular survival, we first quantified expression levels of neuroprotective genes and those associated with the ferroptosis process. We detected 0.63-fold lower *NUBP2* transcript levels in unstressed hNgb-EGFP cells as compared to values of EGFP control cells. NUBP2 is a part of the iron-sulfur protein assembly machinery and polymorphisms within the *NUBP2* locus influence IGF-1 and IGFBP-3 levels [38]. Such a link to IGF-signaling is of interest as the latter plays a key role in the regulation of longevity, i.e., decreased IGF pathway activation increases lifespans [39]. Accordingly, IGF cascades and glucose metabolism dysregulation have recently been linked to the pathophysiology of AD [40,41]. IGF1 also popped up as an upstream regulator of hNgb-linked signaling in both our IPA analyses. Besides IGF1-signaling, canonical pathway analysis revealed other pathways linked to cellular senescence and aging: mTOR signaling (*p*-value of 3.62E-02) and the glycolysis pathway (*p*-value of 4.56E-04). Together with the protein ubiquitination pathway (*p*-value of 6.41E-03) being linked to hNgb, hNgb could be part of the cell’s main mechanisms to confer protection against age-related pathologies, such as AD, cognitive decline, type 2 diabetes, cancer, and immune system decline [42]. In support of this idea, a knockout of *Ngb* has been already detected to induce expression alterations of proteins linked to the glycolytic pathway [43], and recent research starts to ascribe immune-linked functions to Ngb as well. Ngb has been detected to be linked to astroglial antioxidant mechanisms [44], a neuroprotective interaction between mesenchymal stem cells and astrocytes [45] and the expression of inflammatory markers in the tissue surrounding an injury site [46]. In this study, we detected the interaction of hNgb with DHX9 to be reduced under erastin stress. This helicase is known to take part in pyroptosis, a strongly inflammatory form of programmed cell death [47]. We also identified an increased binding of hNgb to ABRAXAS2 in the ferroptotic cells, a deubiquitinating complex involved in interferon-dependent responses [48]. Ferroptosis is indeed a form of cell death with an inflammatory component due to the release of damage-associated molecular patterns, in contrast to the apoptosis process [6].

We provide further evidence for a link between hNgb and astroglia. In addition to an inflammatory function, astroglia are key to the upkeep of nervous system homeostasis, for which they regulate the glutamate cycle and secrete neurotrophic factors. Not only did we detect IGF1 as an upstream regulator in our network, but other growth factors -FGF2 (fibroblast growth factor 2), TGFA (transforming growth factor-alpha), and EGF (epidermal growth factor)- were identified by IPA as well. Hence, the quest for a mechanistic explanation on how Ngb contributes to the beneficial outcome in a plethora of neurodetrimental insults should include cell renewal as well. In accordance, vimentin has been identified as the target molecule in the network of most of the upstream regulators, including IGF1. This class-III intermediate filament promotes axonal growth and branching [49]. It is re-upregulated in regions with plaque pathology, repairing atrophic dendrites and their lost synaptic connections [49]. Of note, we detected the interaction abundance between hNgb and vimentin to be decreased by a mean fold of 0.31 when cells were erastin-stressed. This raises the question as to whether the interaction with hNgb is activating or inhibiting. We also do not exclude that there might be a different binding ratio when cells would be recovering in non-erastin containing medium. In the non-stress conditions, we further detected hNgb to bind to proteins linked to cell division and differentiation (YWHAZ, RPS4X, TRIM28, YBX1, PGK1, RACK1, XRCC6, EWSR1). Upregulated transcription of *NUBP2* in our hNgb-EGFP cells might result in increased binding of katanin-like 2 protein isoforms, which are implicated in cytokinesis and cell cycle progression [50]. Positioning Ngb as such in cell fate pathways is in line with the literature. While Ngb is expressed in embryonic brains and stem cells, its expression is further increased during the differentiation process [51,52]. Closely related to the differentiation mechanism is the process of neuronal sprouting, which occurs during embryogenesis as well as in response to neuronal damage. This study also identified proteins specifically linked to the axonal outgrowth process (YWHAZ, GPI, RACK1, VIM) to bind hNgb. These interactions might be linked to the previously observed positive correlation between Ngb levels and neurite length in Neuro-2a cells [53]. Playing part in the cellular attempt to re-establish tissue homeostasis is further in line with cells to increase their Ngb levels after brain injuries, and this primarily in cells in proximity of the damage [54]. Given the axons of motor- and spinal cord neurons to be the longest in the nervous system, it might be of no surprise that our IPA analysis retrieved motor neuropathies as the highest scoring group in the top of diseases and bio functions. These results are in accordance with Ngb being linked to the SOD1^G93A^ pathology in a mouse model of amyotrophic lateral sclerosis (ALS) [19].

While the aforementioned hNgb-linked regenerative mechanisms could eventually provide a beneficial outcome, the reduction in ROS levels could arrange for the observed direct protection against ferroptosis induction in our hNgb-EGFP SH-SY5Y cells. Mechanisms of action of both erastin and Ngb have indeed been tightly linked with mitochondria. Where erastin has been described to lower mitochondrial volumes, to affect the morphology of the mitochondrial cristae and to promote oxidative stress through voltage-dependent anion channel2/3 [15,16], Ngb has also been described to localize to mitochondria under different conditions [55,56,57,58]. In addition to the well-known ROS/RNS scavenger function of Ngb [37,59], Ngb has been detected to associate with mitochondrial raft-like microdomains where it protects complex IV activity against oxidative stress damage and further acts as a guanine nucleotide dissociation inhibitor for Gα(i/o) [57,58]. Interestingly, we now detected Ngb to bind to HSPB1 (Figure 3, Table S2,S5), which also, at least in part, resides within lipid raft-like domains [60]. HSPB1 is further already known to protect against ferroptotic cell death through the reduction of iron-linked production of lipid ROS [61]. Comparable results were found for hNgb binding to the iron chaperone PCBP3 (Figure 3, Table S2,S5), which binds directly with ferritin [62]. In this study, we also detected a positive correlation between hNgb levels and the expression of *ferritin*, though this correlation did not reach significance. Being an intracellular iron storage protein, elevated ferritin levels can constrain Fenton chemistry and, hence, the formation of free radicals [63]. The importance of ferritin is further substantiated by ferritin knockout mice, of which the brains show increased levels of oxidatively modified proteins and dead cells [64]. Secondly, neuroprotective expression of *NRF2* was increased (2.55 ± 0.36)-fold in erastin-stressed hNgb-EGFP cells, an upregulation which was not seen in EGFP controls. NRF2 is regarded as a negative regulator of ferroptosis and a neuroprotective protein in general [27]. As a way of example, NRF2 induces transcription of radical-detoxifying quinone-1 and is the main regulator of enzymes that are responsible for the production and regeneration of glutathione [27,28,29]. Besides, NRF2 regulates the expression of proteins, which were identified as hNgb binders in this study. It binds antioxidant response elements to increase GPI expression [65] and targets RACK1 [66]. NRF2 itself is regulated by hNgb-binding RNA-binding protein EWS [67]. Hence, this Ngb-EWSR1-NRF2 path could be contributing to the observed upsurge in NRF2 transcripts in our erastin-stressed hNgb-EGFP overexpressing SH-SY5Y cells (Figure 2c, Table S2). Such a link between hNgb and the main regulator of enzymes of the antioxidant glutathione pathway, and the cellular oxidative stress response, in general, is of interest [27,28,29]. It broadens hNgb’s anti-oxidative function beyond its intrinsic scavenger function [30].

In addition to oxidative stress, neurodegenerative pathologies are mostly characterized by deleterious cytosolic and mitochondrial protein aggregates. Ngb is already known to reduce amyloid aggregation [22,68]. In this study, we detected hNgb to be embedded in the protein ubiquitination pathway and the endoplasmic reticulum stress pathway, which is tightly connected to the unfolded protein response [69]. Specifically, we identified multiple proteins of the proteostasis machinery to bind hNgb: RPS4X, HSPA5, GPI, RACK1, UCHL1, HSPB1. The heat shock protein HSPA5 is part of the cellular defense apparatus against protein misfolding with an altered expression in AD [70] and ALS [71]. UCHL1 functions in the autophagy/lysosomal pathway and has been linked to pathologies of both the central and peripheral nervous system [72]. The hNgb-UCHL1-APP axis is further of interest, providing a link with upstream regulator REST/NRSF. This transcription factor represses genes involved in cell death and upsurges expression of neurotransmission-linked genes in the early stages of the AD pathology [21]. Besides, REST-Ngb expression correlations have already been described by us in cortices of APP23 mice [20]. Interestingly, we furthermore detected hNgb to bind to the 14-3-3 protein (YWHAZ). This interaction has also been detected in the only other study on hNgb interactors in a human-derived setting, i.e., SH-SY5Y cells [23]. Jayaraman et al. used a protein sequence model to identify possible protein binding sites on hNgb, out of which 14-3-3 came out as a candidate. The interaction was checked with a Co-IP/WB approach and a FRET analysis [23]. Detecting the interaction in both studies not only contributes to the validity of the other found interactions, but it provides interesting links to other studies and known aspects of Ngb biology. YWHAZ is an adapter protein implicated in intracellular signaling, apoptosis, cell division, and differentiation. Moreover, it is a strong candidate for cross-seeding and interacts with amyloid-β and tau [73]. Transgenic overexpression increases amyloid-β toxicity [73]. Hence, the combination of the hNgb-HSPA5-APP, hNgb-UCHL1-APP, and hNgb-YWHAZ-APP axes could be central to the neuroprotective effect of hNgb towards the amyloid aggregation pathology (Figure 6b).

## 5. Conclusions

This study widened the endogenous neuroprotective actions of hNgb to ferroptotic cell death, detecting 0.68-fold less cell death in erastin-stressed hNgb-EGFP cells. We further described hNgb protein interactions in a human-derived neuronal setting in both a physiological and a ferroptotic condition. The associated pathways highlighted by the IPA analysis provide a better understanding of hNgb’s cellular role and the cellular response towards ferroptotic stress in general. Future work will focus on identifying the activating or inhibitory nature of the interactions, with a special interest towards enzymes linked to both proteostasis and neuron sprouting (e.g., glucose-6-phosphate isomerase). Identifying vimentin as a hub protein in hNgb-linked networks makes further research into this interaction warranted. Also, with mitochondria associating with vimentin intermediate filaments, it would be interesting to investigate the subcellular location (e.g., mitochondrial lipid rafts) where Ngb interacts with vimentin as well as with its other binding partners. 

## Supplementary Materials

The following are available online at https://www.mdpi.com/2073-4409/8/8/873/s1, Figure S1: DNA sequence of the hNgb-EGFP fusion protein, Table S1: Primers used for qPCR analysis, Table S2: Physiological functions of the 24 proteins that bind to hNgb in a non-stress condition; unlabeled Co-IP MS/MS experiment, Table S3: Proteins identified to bind hNgb in only one of the two unstressed hNgb-EGFP samples, but which could be specific as they were not retrieved in the EGFP control samples; unlabeled Co-IP MS/MS experiment, Table S4: Physiological functions of the 10 proteins that were identified in the SILAC experiment as hNgb-binders, Table S5: Proteins identified to bind hNgb in only the forward or reverse SILAC experiment, but which could be specific as they were not retrieved in the EGFP control samples.
