# Supplementary material for: Connecting the Dots in the Neuroglobin-Protein Interaction Network of an Unstressed and Ferroptotic Cell Death Neuroblastoma Model
Source: Cells. 2019 Aug 11;8(8):873. doi: 10.3390/cells8080873 (PMC6721670; doi:10.3390/cells8080873)
Supplement: Supplementary file 1 [file cells-08-00873-s001.pdf]

Supplementary Information

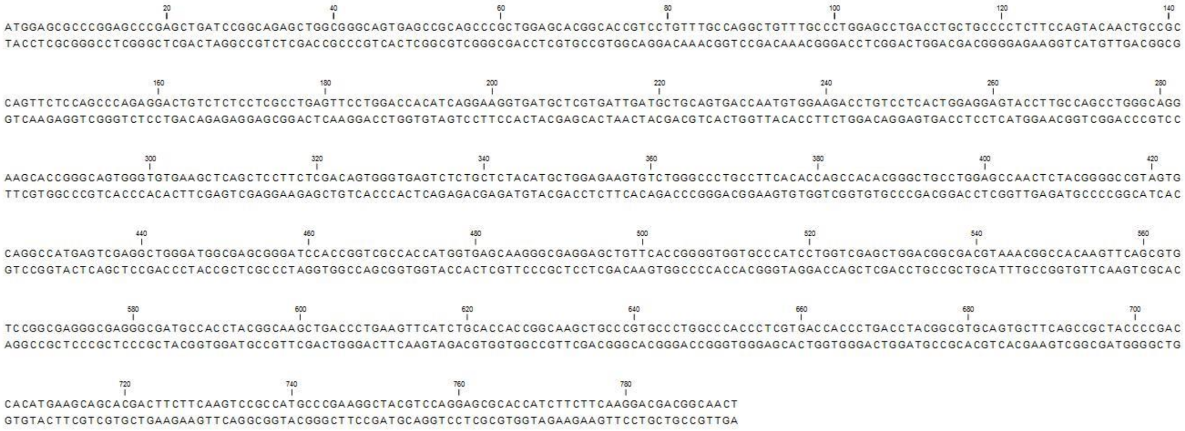

Figure S1. DNA sequence of the hNgb-EGFP fusion protein. Sequence of the coding region of human Ngb (NM_021257.3) ligated in the pEGFP-N1 vector (Clontech), as verified by means of Sanger sequencing.

Table S1. Primers used for qPCR analysis.

Housekeeping genes			
	ACTB	F-GCCGCCAGCTCACCAT	R-TCGATGGGGTACTTCAGGGT
	B2M	F-AGCAGCATCATGGAGGTTTG	R-AGCCCTCCTAGAGCTACCTG
Transcripts of interest			
	CHAC1	F-GAACCCTGGTTACCTGGGC	R-CGCAGCAAGTATTCAAGGTTGT
	Ferritin	F-ATTTCGACCGCGATGATGTGG	R-GAACCCAGGGCATGAAGATCC
	hNgb	F-GAAGCACCGGGCAGTG	R-AGACACTTCTCCAGCATGTAGAG
	HO1	F-CCAGCGGGCCAGCAACAAAGTGC	R-AAGCCTTCAGTGCCCACGGTAAGG
	NRF2	F-TCCAGTCAGAAACCAGTGGAT	R-GAATGTCTGCGCCAAAAGCTG
	NUBP2	F-CAGAGCATCTCGCTCATGTCT	R-TATCAGCGCGTTTTTCTTGGG
	SLC7A11	F-AGCAGCAGCAGCAGTGGT	R-CTGTGTATGCATCGTGCTCTC
	TFR1	F-TCGTGAGGCTGGATCTCAAAA	R-CCTTACTATACGCCACATAACCC

Table S2. Physiological functions of the 24 proteins that bind to hNgb in a non-stress condition; unlabeled Co-IP MS/MS experiment.

**P63104**	**14-3-3 protein zeta/delta**<br>Adapter protein implicated in intracellular signaling, apoptosis, cell division and differentiation. As such the protein has been found implicated in neuronal disorders as Creutzfeldt–Jakob disease, Alzheimer's disease (AD), neuronal migration defects and polyglutamine diseases [1].
**P62701**	**40S ribosomal protein S4, X isoform**<br>Cytoplasmic protein enhancing cell proliferation and translation [2]. The protein is upregulated in plaque tissue of multiple sclerosis [3].
**P08865**	**40S ribosomal protein SA**<br>Levels of this protein are positively associated with cell survival; i.e. both the generation of neoplasms as the protection of neurons against apoptosis-inducing stimuli [4].
**P18124**	**60S ribosomal protein L7**<br>Structural constituent of ribosomes, involved in translation.
**P61313**	**60S ribosomal protein L15**<br>A large deletion in the *RPL15* gene is causative for the development of Diamond-Blackfan anemia 12 [5]. The protein interacts with interferon-inducible protein p56 [6].
**P83881**	**60S ribosomal protein L36a**<br>Structural constituent of ribosomes, involved in translation.
**P11021**	**78 kDa glucose-regulated protein**<br>Cellular defense apparatus against protein misfolding with an altered expression in AD [7] and Amyotrophic Lateral Sclerosis (ALS) [8]. The protein has neurogenic functions as well [9].
**P68032**	**Actin**<br>Actin is a highly conserved protein involved in synaptic dynamics [10] and axon degeneration through caspase-mediated cleavage. The latter may occur during development, the physiological turnover of neurons and neuronal injury [11].
**Q08211**	**ATP-dependent RNA helicase A**<br>Nucleic acid helicase which, in addition, is involved in different forms of cell death: developmental apoptosis of neural progenitors [12] and pyroptosis as a host defense against viral infections [13].
**O75531**	**Barrier-to-autointegration factor**<br>The protein is involved in chromatin decondensation and nuclear growth [14]. A homozygous mutation in *BANF1* causes Nestor-Guillermo progeria syndrome [15].
**P12277**	**Creatine kinase B-type**<br>Energy transfer protein which is upregulated during cerebellar postnatal development [16] and downregulated in AD and schizophrenia [17]. It activates neuron-specific K^+-Cl^- co-transporter KCC2 [17].
**P06744**	**Glucose-6-phosphate isomerase**<br>Glucose metabolism enzyme with a role in proteostasis [18] and motor neuron sprouting [19]. It has more general neurotrophic properties as well [20].
**P04792**	**Heat shock protein beta-1**<br>Molecular chaperone with a role in the anterograde axonal transport of proteins [21] and mitochondria [22], VEGF-mediated angiogenesis [23], oxidative stress regulation, regulation of apoptosis, and the mediation of translational repression [24].
**P01834**	**Immunoglobulin kappa constant**<br>Constant part of antibodies which gets significantly oxidized in AD affected serum [25] and is upregulated in inflammatory neuropathies [26].
**P07195**	**L-lactate dehydrogenase B chain**<br>Part of the fermentation pathway that is involved in the brain energy metabolism [27].
**P67809**	**Nuclease-sensitive element-binding protein 1**<br>YB-1 is a protein with pleiotropic functions e.g. transcriptional regulation, DNA repair, cell proliferation, and stress responses to extracellular signals [28]. Moreover, it influences the pluripotency state of embryonic stem cells [29].
**P00558**	**Phosphoglycerate kinase 1**<br>Glycolytic enzyme of which the overexpression alleviates spinal muscular atrophy phenotypes [30]. It participates in angiogenesis by reducing plasmin [31] and is involved in cellular differentiation [32].
**P57721**	**Poly(rC)-binding protein 3**<br>Alpha-CP3 has a role in post-transcriptional activities and functions as an iron chaperone [33]. Alpha-CP3 binds directly with ferritin and, hence, contributes to the iron deficiency response [33]. Splicing regulation of i.a. Tau, links Alpha-CP3

	to tauopathy dementias [34].
P63244	**Receptor of activated protein C kinase 1**<br>Scaffold protein implicated in axon guidance and outgrowth [35], the protection against oxidative stress-induced apoptosis [36], G0/G1 cell division transitions [37] and the inhibition of phagocytosis [38]. RACK1 may contribute in the development of cytoplasmic inclusions in aggregation pathologies [39].
Q01844	**RNA-binding protein EWS**<br>Transcriptional repressor with a role in stem cell quiescence [40]. Disease-specific variants are aggregation-prone and may lead to ALS pathology [41].
Q13263	**Transcription intermediary factor 1-beta**<br>Scaffold protein which binds the transcription factor REST/NRSF, promoting neuronal differentiation [42]. TIF1-beta further regulates gene transcription through chromatin remodeling [43].
P09936	**Ubiquitin carboxyl-terminal hydrolase isozyme L1**<br>Protein involved in the autophagy/lysosomal pathway [44], maintaining the structure and function of the neuromuscular junction [45] and the physiological working of the endoplasmic reticulum [46]. Mutations in UCHL1 are associated with autosomal dominant Parkinson disease [47] and lowered UCHL1 levels are detected in ALS and AD as well [46].
P08670	**Vimentin**<br>Class-III intermediate filament which promotes axonal growth and branching [48], and negatively regulates peripheral nerve myelination [49]. The protein is re-upregulated in regions with plaque pathology, repairing atrophic dendrites and their lost synaptic connections [48].
P12956	**X-ray repair cross-complementing protein 6**<br>ATP-dependent helicase implicated in embryonic neurogenesis [50] and the production of IFN-λ1 [51]. Its expression levels are further correlated with telomere length [52]. Acetylated Ku70 induces caspase-dependent cell death [53]. It forms a heterodimer with X-ray repair cross-complementing protein 5 [54].

Table S3. Proteins identified to bind hNgb in only one of the two unstressed hNgb-EGFP samples, but which could be specific as they were not retrieved in the EGFP control samples; unlabeled Co-IP MS/MS experiment.

38 proteins identified solely in hNgb-EGFP S1		59 proteins identified solely in hNgb-EGFP S2	
TUBA1C	Tubulin alpha-1C chain	HIST2H2AB	Histone H2A type 2-B
IGLC2	Immunoglobulin lambda constant 2	TUBB4A	Tubulin beta-4A chain
HIST1H1E	Histone H1.4	HIST1H2AA	Histone H2A type 1-A
FUS	RNA-binding protein FUS	CFL1	Cofilin-1
C1QBP	Complement component 1 Q subcomponent-binding protein, mitochondrial	DUT	Deoxyuridine 5'-triphosphate nucleotide-hydrolase, mitochondrial
HNRNPL	Heterogeneous nuclear ribonucleoprotein L	RPL38	60S ribosomal protein L38
ALYREF	THO complex subunit 4	GSTP1	Glutathione S-transferase P
XRCC5	X-ray repair cross-complementing protein 5	PCBP1	Poly(rC)-binding protein 1
EIF4A2	Eukaryotic initiation factor 4A-II	RPL10	60S ribosomal protein L10
HNRNPD	Heterogeneous nuclear ribonucleoprotein D0	HMGB1	High mobility group protein B1
NOLC1	Nucleolar and coiled-body phosphoprotein 1	TUBA4B	Putative tubulin-like protein alpha-4B
GDI1	Rab GDP dissociation inhibitor alpha	HNRNPA3	Heterogeneous nuclear ribonucleoprotein A3
TMPO	Lamina-associated polypeptide 2, alpha	SSBP1	Single-stranded DNA-binding protein
RPLP2	60S acidic ribosomal protein P2	SNU13	NHP2-like protein 1
KIF5B	Kinesin-1 heavy chain	YWHAB	14-3-3 protein beta/alpha
HNRNPR	Heterogeneous nuclear ribonucleoprotein R	TEX264	Testis-expressed protein 264
AHSG	Alpha-2-HS-glycoprotein	RPS3A	40S ribosomal protein S3a
MIF	Macrophage migration inhibitory factor	IGLV3-19	Immunoglobulin lambda variable 3-19
HMGA1	High mobility group protein HMG-I/HMG-Y	DPYSL2	Dihydropyrimidinase-related protein 2
PDIA3	Protein disulfide-isomerase A3	RPL34	60S ribosomal protein L34
MATR3	Matrin-3	WBP11	WW domain-binding protein 11
RPA1	Replication protein A 70 kDa DNA-binding subunit	YWHAE	14-3-3 protein epsilon
RPS7	40S ribosomal protein S7	YWHAG	14-3-3 protein gamma
MKI67	Proliferation marker protein Ki-67	USP44	Ubiquitin carboxyl-terminal hydrolase 44
HNRNPA0	Heterogeneous nuclear ribonucleoprotein A0	RPS13	40S ribosomal protein S13
HSPA9	Stress-70 protein, mitochondrial	KSR2	Kinase suppressor of Ras 2
RPLP1	60S acidic ribosomal protein P1	ENO2	Gamma-enolase
CBX3	Chromobox protein homolog 3	MYL6B	Myosin light chain 6B
ANXA2P2	Putative annexin A2-like protein	EIF4A1	Eukaryotic initiation factor 4A-I
KPNB1	Importin subunit beta-1	FSCN1	Fascin
RPL32	60S ribosomal protein L32	RPL13A	60S ribosomal protein L13a
RPS19	40S ribosomal protein S19	PRDX2	Peroxiredoxin-2
U2AF1L5	Splicing factor U2AF 35 kDa subunit-like protein	KIAA1614	Uncharacterized protein KIAA1614
CRMP1	Dihydropyrimidinase-related protein 1	NAP1L1	Nucleosome assembly protein 1-like 1
SRSF3	Serine/arginine-rich splicing factor 3	RPA2	Replication protein A 32 kDa subunit
TPR	Nucleoprotein TPR	CCT4	T-complex protein 1 subunit delta
UVSSA	UV-stimulated scaffold protein A	PARK7	Protein/nucleic acid deglycase DJ-1
RALGAPA1	Ral GTPase-activating protein subunit alpha-1	PDIA6	Protein disulfide-isomerase A6
		ACTBL2	Beta-actin-like protein 2
		GNB3	Guanine nucleotide-binding protein G(I)/G(S)/G(T) subunit beta-3
		RUVBL1	RuvB-like 1
		RPLP0	60S acidic ribosomal protein P0
		PLXNA4	Plexin-A4
		RPL5	60S ribosomal protein L5
		ATP5B	ATP synthase subunit beta, mitochondrial
		KHDRBS1	KH domain-containing, RNA-binding, signal transduction-associated protein 1
		ATP5A1	ATP synthase subunit alpha, mitochondrial
		ALDOA	Fructose-bisphosphate aldolase A
		CCT6A	T-complex protein 1 subunit zeta
		PDIA4	Protein disulfide-isomerase A4
		CCT8	T-complex protein 1 subunit theta
		CCT3	T-complex protein 1 subunit gamma
		UBA1	Ubiquitin-like modifier-activating enzyme 1
		KBTBD3	Kelch repeat and BTB domain-containing protein 3
		SMC3	Structural maintenance of chromosomes protein 3

	NACA	Nascent polypeptide-associated complex subunit alpha, muscle-specific form
	LRPPRC	Leucine-rich PPR motif-containing protein, mitochondrial
	FASN	Fatty acid synthase
	MUC16	Mucin-16

Table S4. Physiological functions of the 10 proteins that were identified in the SILAC experiment as hNgb-binders.

**P62081**	**40S ribosomal protein S7**<br>Ribosomal protein linked to the cellular apoptosis process through abrogation of oncogene MDM2-mediated p53 ubiquitination [55]. Mutation variants are linked to Diamond-Blackfan anemia [5].
**Q08211**	**ATP-dependent RNA helicase A**<br>Protein detected in both the non-SILAC and SILAC experiment. Nucleic acid helicase which, in addition, is involved in different forms of cell death: developmental apoptosis of neural progenitors [12] and pyroptosis as a host defense against viral infections [13].
**Q15018**	**BRISC complex subunit Abraxas 2**<br>Brcc36-containing isopeptidase complex unit which is involved in deubiquitinating proteins. Through this function it regulates p53 activity [56] and interferon-dependent responses [57].
**Q9NZB2**	**Constitutive coactivator of PPAR-gamma-like protein 1**<br>Scaffold protein involved in phosphorylation cascades of the FAK and PI3K/AKT/mTOR pathways [58], with downstream the Akt-mediated antiapoptotic cascade [59]. It promotes secretion of neurotrophic IGF2 [59] and interacts with kinesins responsible for transporting molecular cargo [58].
**P84090**	**Enhancer of rudimentary homolog**<br>Protein that is linked to mRNA splicing, the replication stress response, the cell cycle, and optimal cell growth under stress conditions [60].
**P51991**	**Heterogeneous nuclear ribonucleoprotein A3**<br>Protein is actively 'recruited' in protein aggregates in C9orf72-linked frontotemporal lobar degeneration and motor neuron disease [61]. Reduced levels of hnRNPA3 induce cellular senescence, e.g. reduction in cell growth, the upsurge in dsDNA breaks, and the increase of p53 and p21/WAF1 proteins [62].
**P52272**	**Heterogeneous nuclear ribonucleoprotein M**<br>Pre-mRNA binding protein linked to spinal muscular atrophy [63]. It also functions as a cell surface adhesion receptor, making it function in an ephrin receptor-like way to regulate axonal targeting [64].
**Q9P0L0**	**Vesicle-associated membrane protein-associated protein A**<br>Integral membrane protein known to function in the regulation of sterol, lipid biosynthesis and vesicle trafficking. It binds the electromotility protein prestin [65] and protrudin [66], making VAPA involved in the neurite outgrowth process. Plays a role in the pathophysiology of amyotrophic lateral sclerosis [67].
**P08670**	**Vimentin**<br>Protein detected in both the non-SILAC and SILAC experiment. Class-III intermediate filament which promotes axonal growth and branching [48], and negatively regulates peripheral nerve myelination [49]. The protein is re-upregulated in regions with plaque pathology, repairing atrophic dendrites and their lost synaptic connections [48].
**P13010**	**X-ray repair cross-complementing protein 5**<br>Helicase of which the levels are positively correlated with longevity [54]. It forms a heterodimer with X-ray repair cross-complementing protein 6 [54]. As such XRCC5 is involved in embryonic neurogenesis [50].

Table S5. Proteins identified to bind hNgb in only the forward or reverse SILAC experiment, but which could be specific as they were not retrieved in the EGFP control samples.

Elements found in either the "Forward" or "Reverse" experiment		Abundances: Forward			Abundances: Reverse		
		Stress	Non-stress	S/NS	Stress	Non-stress	S/NS
RTL1	Retrotransposon-like protein 1	2,28E+05	NA	n.a.	n.a.	n.a.	n.a.
CNBP	Cellular nucleic acid-binding protein	NA	1,56E+06	n.a.	n.a.	n.a.	n.a.
RUFY1	RUN & FYVE domain-containing protein 1	3,96E+07	1,87E+07	2,12	n.a.	n.a.	n.a.
RUFY3	Protein RUFY3	NA	NA	n.a.	n.a.	n.a.	n.a.
PFN2	Profilin-2	2,51E+06	NA	n.a.	n.a.	n.a.	n.a.
HP	Haptoglobin	NA	NA	n.a.	n.a.	n.a.	n.a.
SNRPC	U1 small nuclear ribonucleoprotein C	NA	NA	n.a.	n.a.	n.a.	n.a.
YTHDF3	YTH domain-containing family protein 3	5,95E+06	3,18E+05	18,70	n.a.	n.a.	n.a.
DLST	Dihydrolipoyllysine-residue succinyltransferase component of 2-oxoglutarate dehydrogenase complex	1,67E+06	NA	n.a.	n.a.	n.a.	n.a.
TBC1D5	TBC1 domain family member 5	1,90E+06	1,16E+06	1,64	n.a.	n.a.	n.a.
RPL30	60S ribosomal protein L30	NA	NA	n.a.	n.a.	n.a.	n.a.
VAPB	Vesicle-associated membrane protein-associated protein B/C	3,88E+07	1,79E+07	2,16	n.a.	n.a.	n.a.
WTAP	Pre-mRNA-splicing regulator WTAP	3,15E+06	1,12E+06	2,80	n.a.	n.a.	n.a.
SNRPF	Small nuclear ribonucleoprotein F	NA	NA	n.a.	n.a.	n.a.	n.a.
YBX1	Nuclease-sensitive element-binding protein 1	1,09E+06	4,89E+05	2,23	n.a.	n.a.	n.a.
MT1G	Metallothionein-1G	NA	1,69E+06	n.a.	n.a.	n.a.	n.a.
SNRPD3	Small nuclear ribonucleoprotein Sm D3	1,99E+07	5,54E+06	3,59	n.a.	n.a.	n.a.
HSPA9	Stress-70 protein, mitochondrial	NA	NA	n.a.	n.a.	n.a.	n.a.
SNRPD2	Small nuclear ribonucleoprotein Sm D2	5,46E+06	NA	n.a.	n.a.	n.a.	n.a.
RUVBL1	RuvB-like 1	NA	NA	n.a.	n.a.	n.a.	n.a.
TRIM28	Transcription intermediary factor 1-beta	2,37E+07	7,03E+06	3,38	n.a.	n.a.	n.a.
SNRPA	U1 small nuclear ribonucleoprotein A	4,58E+07	8,71E+06	5,26	n.a.	n.a.	n.a.
SRSF8	Serine/arginine-rich splicing factor 8	NA	NA	n.a.	n.a.	n.a.	n.a.
CCT6A	T-complex protein 1 subunit zeta	3,43E+05	NA	n.a.	n.a.	n.a.	n.a.
IGKV1-5	Immunoglobulin kappa variable 1-5	NA	NA	n.a.	n.a.	n.a.	n.a.
ITIH3	Inter-alpha-trypsin inhibitor heavy chain H3	NA	NA	n.a.	n.a.	n.a.	n.a.
PTBP1	Polypyrimidine tract-binding protein 1	NA	NA	n.a.	n.a.	n.a.	n.a.
CNTNAP4	Contactin-associated protein-like 4	2,49E+07	NA	n.a.	n.a.	n.a.	n.a.
IGHG4	Immunoglobulin heavy constant gamma 4	NA	NA	n.a.	n.a.	n.a.	n.a.
RPLP0	60S acidic ribosomal protein P0	2,58E+06	4,70E+05	5,48	n.a.	n.a.	n.a.
HNRNPL	Heterogeneous nuclear ribonucleoprotein L	7,89E+06	1,36E+06	5,79	n.a.	n.a.	n.a.
TUBA4B	Putative tubulin-like protein alpha-4B	1,53E+07	2,52E+06	6,10	n.a.	n.a.	n.a.
TF	Serotransferrin	2,21E+06	NA	n.a.	n.a.	n.a.	n.a.
HNRNPD	Heterogeneous nuclear ribonucleoprotein D0	1,11E+07	2,31E+06	4,78	n.a.	n.a.	n.a.
CCT5	T-complex protein 1 subunit epsilon	NA	NA	n.a.	n.a.	n.a.	n.a.
CBX3	Chromobox protein homolog 3	NA	NA	n.a.	n.a.	n.a.	n.a.
HIST2H2AB	Histone H2A type 2-B	n.a.	n.a.	n.a.	3,57E+08	1,10E+09	0,32
TUBA1A	Tubulin alpha-1A chain	n.a.	n.a.	n.a.	NA	NA	NA
HSPB1	Heat shock protein beta-1	n.a.	n.a.	n.a.	NA	1,18E+06	n.a.
PCBP3	Poly(rC)-binding protein 3	n.a.	n.a.	n.a.	NA	4,67E+05	n.a.
SNRPD1	Small nuclear ribonucleoprotein Sm D1	n.a.	n.a.	n.a.	NA	NA	n.a.
RPL38	60S ribosomal protein L38	n.a.	n.a.	n.a.	8,00E+05	8,53E+05	0,94
MYH10	Myosin-10	n.a.	n.a.	n.a.	NA	NA	n.a.
MYH9	Myosin-9	n.a.	n.a.	n.a.	NA	NA	n.a.
PDIA3	Protein disulfide-isomerase A3	n.a.	n.a.	n.a.	NA	NA	n.a.
BANF1	Barrier-to-autointegration factor	n.a.	n.a.	n.a.	NA	2,73E+07	n.a.

GPI	Glucose-6-phosphate isomerase	n.a.	n.a.	n.a.	NA	3,80E+05	n.a.
MAP4	Microtubule-associated protein 4	n.a.	n.a.	n.a.	NA	9,15E+05	n.a.
RPL26	60S ribosomal protein L26	n.a.	n.a.	n.a.	NA	NA	n.a.
ALYREF	THO complex subunit 4	n.a.	n.a.	n.a.	NA	1,52E+05	n.a.
SLC25A31	ADP/ATP translocase 4	n.a.	n.a.	n.a.	NA	NA	n.a.
RPL7	60S ribosomal protein L7	n.a.	n.a.	n.a.	NA	NA	n.a.
RPLP2	60S acidic ribosomal protein P2	n.a.	n.a.	n.a.	NA	NA	n.a.
ANXA1	Annexin A1	n.a.	n.a.	n.a.	NA	3,16E+06	n.a.
NOLC1	Nucleolar and coiled-body phosphoprotein 1	n.a.	n.a.	n.a.	5,44E+05	2,92E+05	1,86
HADHA	Trifunctional enzyme subunit α, mitochondrial	n.a.	n.a.	n.a.	NA	NA	n.a.
HSP90B1	Endoplasmin	n.a.	n.a.	n.a.	NA	6,31E+05	n.a.
MYL6B	Myosin light chain 6B	n.a.	n.a.	n.a.	NA	NA	n.a.
SNU13	NHP2-like protein 1	n.a.	n.a.	n.a.	NA	NA	n.a.
XRCC6	X-ray repair cross-complementing protein 6	n.a.	n.a.	n.a.	NA	3,52E+05	n.a.
FASN	Fatty acid synthase	n.a.	n.a.	n.a.	NA	NA	n.a.
HMGN1	Non-histone chromosomal protein HMG-14	n.a.	n.a.	n.a.	NA	NA	n.a.

NA: no abundance value available, i.e. when there is a good MS2 available but the MS1 level peak is not sufficiently reliable for integration (e.g. due to co-elution of another peptide, resulting in "shoulder formation"). n.a. = not applicable

References

1. Berg, D.; Holzmann, C.; Riess, O. 14-3-3 proteins in the nervous system. *Nat Rev Neurosci* **2003**, *4*, 752-762, doi:10.1038/nrn1197.
2. Watanabe, M.; Furuno, N.; Goebl, M.; Go, M.; Miyauchi, K.; Sekiguchi, T.; Basilico, C.; Nishimito, T. Molecular cloning of the human gene, CCG2, that complements the BHK-derived temperature-sensitive cell cycle mutant tsBN63: identity of CCG2 with the human X chromosomal SCAR/RPS4X gene. *J Cell Sci* **1991**, *100 (Pt 1)*, 35-43.
3. Tajouri, L.; Mellick, A.S.; Ashton, K.J.; Tannenberg, A.E.; Nagra, R.M.; Tourtellotte, W.W.; Griffiths, L.R. Quantitative and qualitative changes in gene expression patterns characterize the activity of plaques in multiple sclerosis. *Brain Res Mol Brain Res* **2003**, *119*, 170-183.
4. Meloni, B.P.; Tilbrook, P.A.; Boulos, S.; Arthur, P.G.; Knuckey, N.W. Erythropoietin preconditioning in neuronal cultures: signaling, protection from in vitro ischemia, and proteomic analysis. *J Neurosci Res* **2006**, *83*, 584-593, doi:10.1002/jnr.20755.
5. Landowski, M.; O'Donohue, M.F.; Buros, C.; Ghazvinian, R.; Montel-Lehry, N.; Vlachos, A.; Sieff, C.A.; Newburger, P.E.; Niewiadomska, E.; Matysiak, M., et al. Novel deletion of RPL15 identified by array-comparative genomic hybridization in Diamond-Blackfan anemia. *Hum Genet* **2013**, *132*, 1265-1274, doi:10.1007/s00439-013-1326-z.
6. Hsu, Y.A.; Lin, H.J.; Sheu, J.J.; Shieh, F.K.; Chen, S.Y.; Lai, C.H.; Tsai, F.J.; Wan, L.; Chen, B.H. A novel interaction between interferon-inducible protein p56 and ribosomal protein L15 in gastric cancer cells. *DNA Cell Biol* **2011**, *30*, 671-679, doi:10.1089/dna.2010.1149.
7. Chen, S.; Lu, F.F.; Seeman, P.; Liu, F. Quantitative proteomic analysis of human substantia nigra in Alzheimer's disease, Huntington's disease and Multiple sclerosis. *Neurochem Res* **2012**, *37*, 2805-2813, doi:10.1007/s11064-012-0874-2.
8. Filareti, M.; Luotti, S.; Pasetto, L.; Pignataro, M.; Paolella, K.; Messina, P.; Pupillo, E.; Filosto, M.; Lunetta, C.; Mandrioli, J., et al. Decreased Levels of Foldase and Chaperone Proteins Are Associated with an Early-Onset Amyotrophic Lateral Sclerosis. *Front Mol Neurosci* **2017**, *10*, 99, doi:10.3389/fnmol.2017.00099.
9. Bestman, J.E.; Huang, L.C.; Lee-Osbourne, J.; Cheung, P.; Cline, H.T. An in vivo screen to identify candidate neurogenic genes in the developing Xenopus visual system. *Dev Biol* **2015**, *408*, 269-291, doi:10.1016/j.ydbio.2015.03.010.
10. D'Ambrosi, N.; Rossi, S.; Gerbino, V.; Cozzolino, M. Rac1 at the crossroad of actin dynamics and neuroinflammation in Amyotrophic Lateral Sclerosis. *Front Cell Neurosci* **2014**, *8*, 279, doi:10.3389/fncel.2014.00279.
11. Sokolowski, J.D.; Gamage, K.K.; Heffron, D.S.; Leblanc, A.C.; Deppmann, C.D.; Mandell, J.W. Caspase-mediated cleavage of actin and tubulin is a common feature and sensitive marker of axonal degeneration in neural development and injury. *Acta Neuropathol Commun* **2014**, *2*, 16, doi:10.1186/2051-5960-2-16.
12. Chen, Z.X.; Wallis, K.; Fell, S.M.; Sobrado, V.R.; Hemmer, M.C.; Ramskold, D.; Hellman, U.; Sandberg, R.; Kenchappa, R.S.; Martinson, T., et al. RNA helicase A is a downstream mediator of KIF1Bbeta tumor-suppressor function in neuroblastoma. *Cancer Discov* **2014**, *4*, 434-451, doi:10.1158/2159-8290.CD-13-0362.
13. Zhu, S.; Ding, S.; Wang, P.; Wei, Z.; Pan, W.; Palm, N.W.; Yang, Y.; Yu, H.; Li, H.B.; Wang, G., et al. Nlrp9b inflammasome restricts rotavirus infection in intestinal epithelial cells. *Nature* **2017**, *546*, 667-670, doi:10.1038/nature22967.
14. Segura-Totten, M.; Kowalski, A.K.; Craigie, R.; Wilson, K.L. Barrier-to-autointegration factor: major roles in chromatin decondensation and nuclear assembly. *J Cell Biol* **2002**, *158*, 475-485, doi:10.1083/jcb.200202019.
15. Puente, X.S.; Quesada, V.; Osorio, F.G.; Cabanillas, R.; Cadinanos, J.; Fraile, J.M.; Ordonez, G.R.; Puente, D.A.; Gutierrez-Fernandez, A.; Fanjul-Fernandez, M., et al. Exome sequencing and functional analysis identifies BANF1 mutation as the cause of a hereditary progeroid syndrome. *Am J Hum Genet* **2011**, *88*, 650-656, doi:10.1016/j.ajhg.2011.04.010.
16. Shen, W.; Willis, D.; Zhang, Y.; Schlattner, U.; Wallimann, T.; Molloy, G.R. Expression of creatine kinase isoenzyme genes during postnatal development of rat brain cerebellum: evidence for transcriptional regulation. *Biochem J* **2002**, *367*, 369-380, doi:10.1042/BJ20020709.
17. Inoue, K.; Yamada, J.; Ueno, S.; Fukuda, A. Brain-type creatine kinase activates neuron-specific K+-Cl-co-transporter KCC2. *J Neurochem* **2006**, *96*, 598-608, doi:10.1111/j.1471-4159.2005.03560.x.
18. Knight, A.L.; Yan, X.; Hamamichi, S.; Ajjuri, R.R.; Mazzulli, J.R.; Zhang, M.W.; Daigle, J.G.; Zhang, S.; Borom, A.R.; Roberts, L.R., et al. The glycolytic enzyme, GPI, is a functionally conserved modifier of

dopaminergic neurodegeneration in Parkinson's models. *Cell Metab* **2014**, *20*, 145-157, doi:10.1016/j.cmet.2014.04.017.
19. English, A.W. Cytokines, growth factors and sprouting at the neuromuscular junction. *J Neurocytol* **2003**, *32*, 943-960, doi:10.1023/B:NEUR.0000020634.59639.cf.
20. Kugler, W.; Breme, K.; Laspe, P.; Muirhead, H.; Davies, C.; Winkler, H.; Schroter, W.; Lakomek, M. Molecular basis of neurological dysfunction coupled with haemolytic anaemia in human glucose-6-phosphate isomerase (GPI) deficiency. *Hum Genet* **1998**, *103*, 450-454.
21. Holmgren, A.; Bouhy, D.; De Winter, V.; Asselbergh, B.; Timmermans, J.P.; Irobi, J.; Timmerman, V. Charcot-Marie-Tooth causing HSPB1 mutations increase Cdk5-mediated phosphorylation of neurofilaments. *Acta Neuropathol* **2013**, *126*, 93-108, doi:10.1007/s00401-013-1133-6.
22. Kalmar, B.; Innes, A.; Wanisch, K.; Kolaszynska, A.K.; Pandraud, A.; Kelly, G.; Abramov, A.Y.; Reilly, M.M.; Schiavo, G.; Greensmith, L. Mitochondrial deficits and abnormal mitochondrial retrograde axonal transport play a role in the pathogenesis of mutant Hsp27-induced Charcot Marie Tooth Disease. *Hum Mol Genet* **2017**, *26*, 3313-3326, doi:10.1093/hmg/ddx216.
23. Lee, Y.J.; Lee, H.J.; Choi, S.H.; Jin, Y.B.; An, H.J.; Kang, J.H.; Yoon, S.S.; Lee, Y.S. Soluble HSPB1 regulates VEGF-mediated angiogenesis through their direct interaction. *Angiogenesis* **2012**, *15*, 229-242, doi:10.1007/s10456-012-9255-3.
24. Geuens, T.; De Winter, V.; Rajan, N.; Achsel, T.; Mateiu, L.; Almeida-Souza, L.; Asselbergh, B.; Bouhy, D.; Auer-Grumbach, M.; Bagni, C., et al. Mutant HSPB1 causes loss of translational repression by binding to PCBP1, an RNA binding protein with a possible role in neurodegenerative disease. *Acta Neuropathol Commun* **2017**, *5*, 5, doi:10.1186/s40478-016-0407-3.
25. Shen, L.; Chen, Y.; Yang, A.; Chen, C.; Liao, L.; Li, S.; Ying, M.; Tian, J.; Liu, Q.; Ni, J. Redox Proteomic Profiling of Specifically Carbonylated Proteins in the Serum of Triple Transgenic Alzheimer's Disease Mice. *Int J Mol Sci* **2016**, *17*, 469, doi:10.3390/ijms17040469.
26. Steck, A.J.; Kinter, J.; Renaud, S. Differential gene expression in nerve biopsies of inflammatory neuropathies. *J Peripher Nerv Syst* **2011**, *16 Suppl 1*, 30-33, doi:10.1111/j.1529-8027.2011.00302.x.
27. Duka, T.; Collins, Z.; Anderson, S.M.; Raghanti, M.A.; Ely, J.J.; Hof, P.R.; Wildman, D.E.; Goodman, M.; Grossman, L.I.; Sherwood, C.C. Divergent lactate dehydrogenase isoenzyme profile in cellular compartments of primate forebrain structures. *Mol Cell Neurosci* **2017**, *82*, 137-142, doi:10.1016/j.mcn.2017.04.007.
28. Kohno, K.; Izumi, H.; Uchiumi, T.; Ashizuka, M.; Kuwano, M. The pleiotropic functions of the Y-box-binding protein, YB-1. *Bioessays* **2003**, *25*, 691-698, doi:10.1002/bies.10300.
29. Guo, C.; Xue, Y.; Yang, G.; Yin, S.; Shi, W.; Cheng, Y.; Yan, X.; Fan, S.; Zhang, H.; Zeng, F. Nanog RNA-binding proteins YBX1 and ILF3 affect pluripotency of embryonic stem cells. *Cell Biol Int* **2016**, *40*, 847-860, doi:10.1002/cbin.10539.
30. Boyd, P.J.; Tu, W.Y.; Shorrock, H.K.; Groen, E.J.N.; Carter, R.N.; Powis, R.A.; Thomson, S.R.; Thomson, D.; Graham, L.C.; Motyl, A.A.L., et al. Bioenergetic status modulates motor neuron vulnerability and pathogenesis in a zebrafish model of spinal muscular atrophy. *PLoS Genet* **2017**, *13*, e1006744, doi:10.1371/journal.pgen.1006744.
31. Lay, A.J.; Jiang, X.M.; Kisker, O.; Flynn, E.; Underwood, A.; Condron, R.; Hogg, P.J. Phosphoglycerate kinase acts in tumour angiogenesis as a disulphide reductase. *Nature* **2000**, *408*, 869-873, doi:10.1038/35048596.
32. Buhrke, T.; Lengler, I.; Lampen, A. Analysis of proteomic changes induced upon cellular differentiation of the human intestinal cell line Caco-2. *Dev Growth Differ* **2011**, *53*, 411-426, doi:10.1111/j.1440-169X.2011.01258.x.
33. Leidgens, S.; Bullough, K.Z.; Shi, H.; Li, F.; Shakoury-Elizeh, M.; Yabe, T.; Subramanian, P.; Hsu, E.; Natarajan, N.; Nandal, A., et al. Each member of the poly-r(C)-binding protein 1 (PCBP) family exhibits iron chaperone activity toward ferritin. *J Biol Chem* **2013**, *288*, 17791-17802, doi:10.1074/jbc.M113.460253.
34. Wang, Y.; Gao, L.; Tse, S.W.; Andreadis, A. Heterogeneous nuclear ribonucleoprotein E3 modestly activates splicing of tau exon 10 via its proximal downstream intron, a hotspot for frontotemporal dementia mutations. *Gene* **2010**, *451*, 23-31, doi:10.1016/j.gene.2009.11.006.
35. Kershner, L.; Welshhans, K. RACK1 regulates neural development. *Neural Regen Res* **2017**, *12*, 1036-1039, doi:10.4103/1673-5374.211175.
36. Ma, J.; Wu, R.; Zhang, Q.; Wu, J.B.; Lou, J.; Zheng, Z.; Ding, J.Q.; Yuan, Z. DJ-1 interacts with RACK1 and protects neurons from oxidative-stress-induced apoptosis. *Biochem J* **2014**, *462*, 489-497, doi:10.1042/BJ20140235.
37. Chang, B.Y.; Conroy, K.B.; Machleder, E.M.; Cartwright, C.A. RACK1, a receptor for activated C kinase and a homolog of the beta subunit of G proteins, inhibits activity of src tyrosine kinases and growth of NIH 3T3 cells. *Mol Cell Biol* **1998**, *18*, 3245-3256.

38. Thorslund, S.E.; Edgren, T.; Pettersson, J.; Nordfelth, R.; Sellin, M.E.; Ivanova, E.; Francis, M.S.; Isaksson, E.L.; Wolf-Watz, H.; Fallman, M. The RACK1 signaling scaffold protein selectively interacts with Yersinia pseudotuberculosis virulence function. *PLoS One* **2011**, *6*, e16784, doi:10.1371/journal.pone.0016784.
39. Russo, A.; Scardigli, R.; La Regina, F.; Murray, M.E.; Romano, N.; Dickson, D.W.; Wolozin, B.; Cattaneo, A.; Ceci, M. Increased cytoplasmic TDP-43 reduces global protein synthesis by interacting with RACK1 on polyribosomes. *Hum Mol Genet* **2017**, *26*, 1407-1418, doi:10.1093/hmg/ddx035.
40. Cho, J.; Shen, H.; Yu, H.; Li, H.; Cheng, T.; Lee, S.B.; Lee, B.C. Ewing sarcoma gene Ews regulates hematopoietic stem cell senescence. *Blood* **2011**, *117*, 1156-1166, doi:10.1182/blood-2010-04-279349.
41. Couthouis, J.; Hart, M.P.; Erion, R.; King, O.D.; Diaz, Z.; Nakaya, T.; Ibrahim, F.; Kim, H.J.; Mojsilovic-Petrovic, J.; Panossian, S., et al. Evaluating the role of the FUS/TLS-related gene EWSR1 in amyotrophic lateral sclerosis. *Hum Mol Genet* **2012**, *21*, 2899-2911, doi:10.1093/hmg/dds116.
42. Lee, N.; Park, S.J.; Haddad, G.; Kim, D.K.; Park, S.M.; Park, S.K.; Choi, K.Y. Interactomic analysis of REST/NRSF and implications of its functional links with the transcription suppressor TRIM28 during neuronal differentiation. *Sci Rep* **2016**, *6*, 39049, doi:10.1038/srep39049.
43. Jakobsson, J.; Cordero, M.I.; Bisaz, R.; Groner, A.C.; Busskamp, V.; Bensadoun, J.C.; Cammas, F.; Losson, R.; Mansuy, I.M.; Sandi, C., et al. KAP1-mediated epigenetic repression in the forebrain modulates behavioral vulnerability to stress. *Neuron* **2008**, *60*, 818-831, doi:10.1016/j.neuron.2008.09.036.
44. Costes, S.; Gurlo, T.; Rivera, J.F.; Butler, P.C. UCHL1 deficiency exacerbates human islet amyloid polypeptide toxicity in beta-cells: evidence of interplay between the ubiquitin/proteasome system and autophagy. *Autophagy* **2014**, *10*, 1004-1014, doi:10.4161/auto.28478.
45. Chen, F.; Sugiura, Y.; Myers, K.G.; Liu, Y.; Lin, W. Ubiquitin carboxyl-terminal hydrolase L1 is required for maintaining the structure and function of the neuromuscular junction. *Proc Natl Acad Sci U S A* **2010**, *107*, 1636-1641, doi:10.1073/pnas.0911516107.
46. Jara, J.H.; Genc, B.; Cox, G.A.; Bohn, M.C.; Roos, R.P.; Macklis, J.D.; Ulupinar, E.; Ozdinler, P.H. Corticospinal Motor Neurons Are Susceptible to Increased ER Stress and Display Profound Degeneration in the Absence of UCHL1 Function. *Cereb Cortex* **2015**, *25*, 4259-4272, doi:10.1093/cercor/bhu318.
47. Leroy, E.; Boyer, R.; Auburger, G.; Leube, B.; Ulm, G.; Mezey, E.; Harta, G.; Brownstein, M.J.; Jonnalagada, S.; Chernova, T., et al. The ubiquitin pathway in Parkinson's disease. *Nature* **1998**, *395*, 451-452, doi:10.1038/26652.
48. Levin, E.C.; Acharya, N.K.; Sedeyn, J.C.; Venkataraman, V.; D'Andrea, M.R.; Wang, H.Y.; Nagele, R.G. Neuronal expression of vimentin in the Alzheimer's disease brain may be part of a generalized dendritic damage-response mechanism. *Brain Res* **2009**, *1298*, 194-207, doi:10.1016/j.brainres.2009.08.072.
49. Triolo, D.; Dina, G.; Taveggia, C.; Vaccari, I.; Porrello, E.; Rivellini, C.; Domi, T.; La Marca, R.; Cerri, F.; Bolino, A., et al. Vimentin regulates peripheral nerve myelination. *Development* **2012**, *139*, 1359-1367, doi:10.1242/dev.072371.
50. Gu, Y.; Sekiguchi, J.; Gao, Y.; Dikkes, P.; Frank, K.; Ferguson, D.; Hasty, P.; Chun, J.; Alt, F.W. Defective embryonic neurogenesis in Ku-deficient but not DNA-dependent protein kinase catalytic subunit-deficient mice. *Proc Natl Acad Sci U S A* **2000**, *97*, 2668-2673.
51. Sui, H.; Zhou, M.; Imamichi, H.; Jiao, X.; Sherman, B.T.; Lane, H.C.; Imamichi, T. STING is an essential mediator of the Ku70-mediated production of IFN-lambda1 in response to exogenous DNA. *Sci Signal* **2017**, *10*, doi:10.1126/scisignal.aah5054.
52. Rutten, E.P.; Gopal, P.; Wouters, E.F.; Franssen, F.M.; Hageman, G.J.; Vanfleteren, L.E.; Spruit, M.A.; Reynaert, N.L. Various Mechanistic Pathways Representing the Aging Process Are Altered in COPD. *Chest* **2016**, *149*, 53-61, doi:10.1378/chest.15-0645.
53. Subramanian, C.; Opipari, A.W., Jr.; Bian, X.; Castle, V.P.; Kwok, R.P. Ku70 acetylation mediates neuroblastoma cell death induced by histone deacetylase inhibitors. *Proc Natl Acad Sci U S A* **2005**, *102*, 4842-4847, doi:10.1073/pnas.0408351102.
54. Lorenzini, A.; Johnson, F.B.; Oliver, A.; Tresini, M.; Smith, J.S.; Hdeib, M.; Sell, C.; Cristofalo, V.J.; Stamato, T.D. Significant correlation of species longevity with DNA double strand break recognition but not with telomere length. *Mech Ageing Dev* **2009**, *130*, 784-792, doi:10.1016/j.mad.2009.10.004.
55. Chen, D.; Zhang, Z.; Li, M.; Wang, W.; Li, Y.; Rayburn, E.R.; Hill, D.L.; Wang, H.; Zhang, R. Ribosomal protein S7 as a novel modulator of p53-MDM2 interaction: binding to MDM2, stabilization of p53 protein, and activation of p53 function. *Oncogene* **2007**, *26*, 5029-5037, doi:10.1038/sj.onc.1210327.
56. Zhang, J.; Cao, M.; Dong, J.; Li, C.; Xu, W.; Zhan, Y.; Wang, X.; Yu, M.; Ge, C.; Ge, Z., et al. ABRO1 suppresses tumourigenesis and regulates the DNA damage response by stabilizing p53. *Nat Commun* **2014**, *5*, 5059, doi:10.1038/ncomms6059.

57. Zeqiraj, E.; Tian, L.; Piggott, C.A.; Pillon, M.C.; Duffy, N.M.; Ceccarelli, D.F.; Keszei, A.F.; Lorenzen, K.; Kurinov, I.; Orlicky, S., et al. Higher-Order Assembly of BRCC36-KIAA0157 Is Required for DUB Activity and Biological Function. *Mol Cell* **2015**, *59*, 970-983, doi:10.1016/j.molcel.2015.07.028.
58. Bartolome, R.A.; Garcia-Palmero, I.; Torres, S.; Lopez-Lucendo, M.; Balyasnikova, I.V.; Casal, J.I. IL13 Receptor alpha2 Signaling Requires a Scaffold Protein, FAM120A, to Activate the FAK and PI3K Pathways in Colon Cancer Metastasis. *Cancer Res* **2015**, *75*, 2434-2444, doi:10.1158/0008-5472.CAN-14-3650.
59. Tanaka, M.; Sasaki, K.; Kamata, R.; Hoshino, Y.; Yanagihara, K.; Sakai, R. A novel RNA-binding protein, Ossa/C9orf10, regulates activity of Src kinases to protect cells from oxidative stress-induced apoptosis. *Mol Cell Biol* **2009**, *29*, 402-413, doi:10.1128/MCB.01035-08.
60. Kavanaugh, G.; Zhao, R.; Guo, Y.; Mohni, K.N.; Glick, G.; Lacy, M.E.; Hutson, M.S.; Ascano, M.; Cortez, D. Enhancer of Rudimentary Homolog Affects the Replication Stress Response through Regulation of RNA Processing. *Mol Cell Biol* **2015**, *35*, 2979-2990, doi:10.1128/MCB.01276-14.
61. Davidson, Y.S.; Flood, L.; Robinson, A.C.; Nihei, Y.; Mori, K.; Rollinson, S.; Richardson, A.; Benson, B.C.; Jones, M.; Snowden, J.S., et al. Heterogeneous ribonuclear protein A3 (hnRNP A3) is present in dipeptide repeat protein containing inclusions in Frontotemporal Lobar Degeneration and Motor Neurone disease associated with expansions in C9orf72 gene. *Acta Neuropathol Commun* **2017**, *5*, 31, doi:10.1186/s40478-017-0437-5.
62. Comegna, M.; Succoio, M.; Napolitano, M.; Vitale, M.; D'Ambrosio, C.; Scaloni, A.; Passaro, F.; Zambrano, N.; Cimino, F.; Faraonio, R. Identification of miR-494 direct targets involved in senescence of human diploid fibroblasts. *FASEB J* **2014**, *28*, 3720-3733, doi:10.1096/fj.13-239129.
63. Cho, S.; Moon, H.; Loh, T.J.; Oh, H.K.; Choy, H.E.; Song, W.K.; Chun, J.S.; Zheng, X.; Shen, H. hnRNP M facilitates exon 7 inclusion of SMN2 pre-mRNA in spinal muscular atrophy by targeting an enhancer on exon 7. *Biochim Biophys Acta* **2014**, *1839*, 306-315, doi:10.1016/j.bbagrm.2014.02.006.
64. Lee, A.R.; Lamb, R.R.; Chang, J.H.; Erdmann-Gilmore, P.; Lichti, C.F.; Rohrs, H.W.; Malone, J.P.; Wairkar, Y.P.; DiAntonio, A.; Townsend, R.R., et al. Identification of potential mediators of retinotopic mapping: a comparative proteomic analysis of optic nerve from WT and Phr1 retinal knockout mice. *J Proteome Res* **2012**, *11*, 5515-5526, doi:10.1021/pr300767a.
65. Sengupta, S.; Miller, K.K.; Homma, K.; Edge, R.; Cheatham, M.A.; Dallos, P.; Zheng, J. Interaction between the motor protein prestin and the transporter protein VAPA. *Biochim Biophys Acta* **2010**, *1803*, 796-804, doi:10.1016/j.bbamcr.2010.03.017.
66. Saita, S.; Shirane, M.; Natume, T.; Iemura, S.; Nakayama, K.I. Promotion of neurite extension by protrudin requires its interaction with vesicle-associated membrane protein-associated protein. *J Biol Chem* **2009**, *284*, 13766-13777, doi:10.1074/jbc.M807938200.
67. Deidda, I.; Galizzi, G.; Passantino, R.; Cascio, C.; Russo, D.; Colletti, T.; La Bella, V.; Guarneri, P. Expression of vesicle-associated membrane-protein-associated protein B cleavage products in peripheral blood leukocytes and cerebrospinal fluid of patients with sporadic amyotrophic lateral sclerosis. *Eur J Neurol* **2014**, *21*, 478-485, doi:10.1111/ene.12334.
